# Oligodendrocyte precursor cell AMPA receptors differ with age and brain region while kainate receptors remain stable

**DOI:** 10.1016/j.isci.2025.113560

**Published:** 2025-09-13

**Authors:** Yasmine Kamen, Kimberley Anne Evans, Yan Ting Ng, Sabine Dietmann, Ragnhildur Thóra Káradóttir

**Affiliations:** 1 Cambridge Stem Cell Institute & Department of Veterinary Medicine, University of Cambridge, Cambridge, UK; 2 Institute for Informatics, Washington University School of Medicine, St. Louis, MO, USA; 3 Department of Physiology, BioMedical Center, Faculty of Medicine, University of Iceland, Reykjavík, Iceland

**Keywords:** Cell biology, Neuroscience

## Abstract

Oligodendrocyte precursor cells (OPCs) proliferate and differentiate into myelinating oligodendrocytes throughout life. Many cues modulate OPC proliferation and differentiation, including neuronal activity, which OPCs sense through voltage-gated ion channels and glutamate receptors. However, OPCs display regional and temporal diversity in the membrane surface expression of these channels and receptors, altering their capacity to sense and respond to neuronal activity. Here, we use whole-cell patch-clamp in acute brain slices and bath-apply 3 μM kainate or 10 μM AMPA to investigate the heterogeneity in kainate and AMPA receptor membrane surface expression. We find that, while kainate receptor current density remains stable with age, OPCs do not respond to neuronal kainate receptor-specific drugs. In contrast, AMPA receptor current density differs with age and between regions, likely due to altered Ca^2+^ permeability and receptor desensitization. The temporal changes in AMPA-evoked currents in OPCs correlate with reported age-related changes in proliferation and differentiation potential.

## Highlights

• Kainate receptor current density is stable with age and between regions in OPCs
• AMPA receptors differ with age and region in OPCs
• AMPA receptor Ca^2+^ permeability increases with age in cortical OPCs
• AMPA receptor desensitization decreases with age in cortical OPCs

## Graphical abstract

**Figure:**
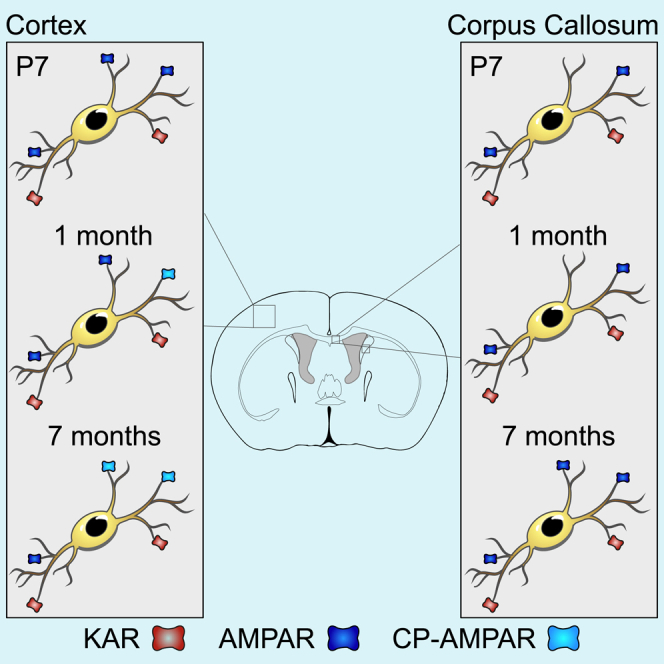

## Introduction

Oligodendrocyte precursor cells (OPCs) proliferate throughout life and can give rise to myelinating oligodendrocytes in the adult central nervous system.^1^ The ongoing differentiation of new oligodendrocytes has been implicated in homeostatic myelin maintenance,^2^ repair following injury,^3^ and myelin plasticity following motor and cognitive learning^4^^,^^5^^,^^6^^,^^7^^,^^8^ and is modulated by sensory stimulation^9^^,^^10^^,^^11^ or social isolation.^12^^,^^13^ Collectively, these studies suggest that neuronal activity may regulate OPC fate, in line with reports that increasing activity with optogenetics or chemogenetics promotes proliferation and differentiation,^14^^,^^15^ while blocking activity with tetrodotoxin or reducing activity by overexpressing Kir2.1 in neurons diminishes proliferation and differentiation.^14^^,^^16^

OPCs express functional voltage-gated ion channels and neurotransmitter receptors, allowing them to sense and respond to neuronal activity.^17^^,^^18^^,^^19^ However, recent evidence suggests that OPC membrane properties differ within and between brain regions and with age, altering OPCs’ sensitivity to neuronal activity, and that this diversity might correlate with OPCs’ proliferation and differentiation potential.^20^^,^^21^^,^^22^^,^^23^ For instance, α-amino-3-hydroxy-5-methyl-4-isoxazolepropionic acid/kainate receptor (AMPAR/KAR) density increases with age and is higher in the cortex than in the corpus callosum, coinciding with reduced proliferation and differentiation rates.^1^^,^^20^ This is in line with reports that activating AMPARs *in vitro* reduces proliferation and differentiation of OPCs^24^^,^^25^^,^^26^ but conflicts with evidence that blocking AMPARs/KARs in demyelinating lesions blocks myelin repair^27^ or that germline AMPAR knockouts do not affect OPC proliferation or differentiation.^28^ These discrepant results might be due to the timing of interventions or differences in functional AMPAR/KAR expression between regions and timepoints, but might also result from modulating only AMPARs or both AMPARs and KARs.

OPCs express both AMPARs and KARs.^28^^,^^29^^,^^30^^,^^31^^,^^32^ However, while many groups have investigated AMPAR signaling in OPCs, very little is known about OPC KARs. In addition, most studies investigating functional expression of AMPARs or KARs alone have focused on a single region and been restricted to single timepoints. While we previously reported that AMPAR/KAR density increases with age and differs between regions,^20^ we used a kainate concentration that activates both non-N-methyl-D-aspartate (NMDA) receptors (AMPARs and KARs),^33^ and thus, whether OPCs exhibit temporal and regional diversity in AMPARs or KARs, or both, remains unclear.

Here we use whole-cell patch-clamp in acute brain slices from heterozygous NG2-EYFP mice^34^ to examine AMPAR and KAR steady-state current density in OPCs between embryonic day 13 (E13) and 7 months in the cortex and corpus callosum. In these mice, EYFP is knocked into the *Cspg4* gene and is therefore tightly coupled to endogenous NG2 expression, allowing unbiased sampling of OPCs. Using 3 μM kainate and 10 μM AMPA to activate KAR and AMPAR, respectively, we find that, while KAR steady-state current density does not differ postnatally with age or region, AMPAR steady-state current density increases with age at different rates between regions. We further examine whether this increase could be explained by differences in Ca^2+^ permeability or subunit splice variants, two determinants of conductance.^35^^,^^36^ We find that Ca^2+^ permeability and decreased desensitization, but not flip/flop splice variants, may contribute to the change in AMPAR current density. These changes correlate with the previously reported proliferation and differentiation potential of OPCs, perhaps underlying cell fate.

## Results

### OPCs have functional kainate receptors

We previously reported that glutamate receptor current densities differ regionally and temporally in OPCs.^20^ Specifically, we found that both NMDAR (activated by 60 μM NMDA) and non-NMDAR (activated with 30 μM kainate) current densities differ between brain regions and with age.^20^ However, as 30 μM kainate activates both AMPARs and KARs,^33^ and OPCs likely express both receptors *in vivo,*^28^^,^^29^^,^^30^^,^^32^ we sought to determine whether AMPARs, KARs, or both differ regionally and temporally.

**Figure 1:**
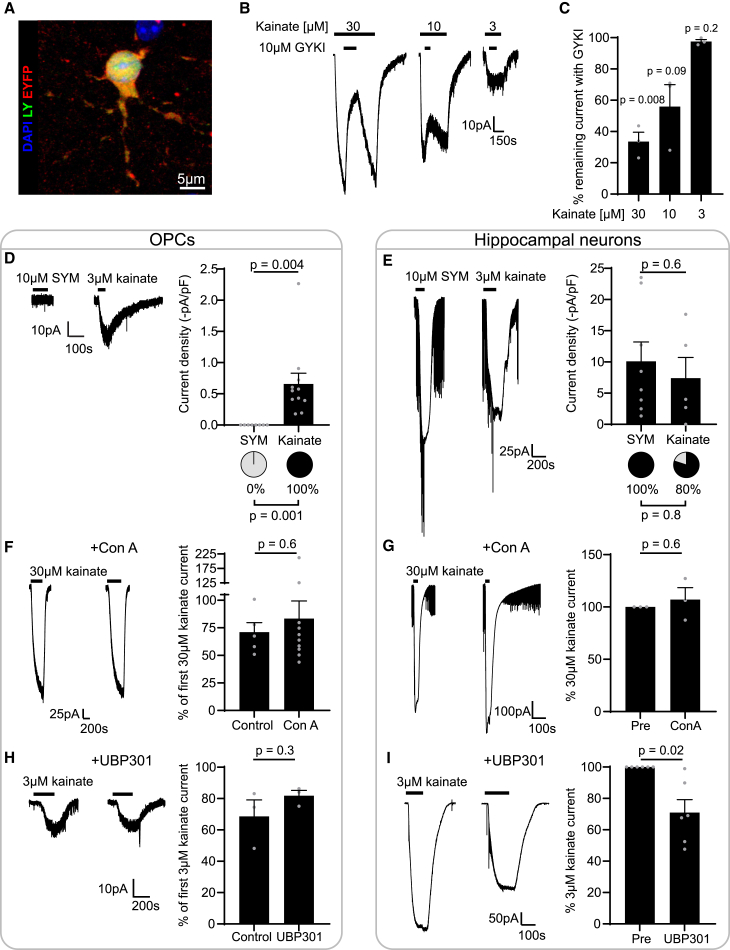
OPC KAR may differ from neuronal KAR (A) Rendering of an EYFP^+^ OPC. EYFP^+^ OPCs were whole-cell patch-clamped in the cortex and corpus callosum and dye-filled with Alexa 594 or Lucifer Yellow (LY). Cells were post hoc immunolabelled against EYFP. Scale bar, 5μm. (B) GYKI53655 (GYKI), 10 μM, an AMPAR-specific antagonist, was applied during 30 μM, 10 μM, or 3 μM kainate application. (C) GYKI blocked 66% of 30 μM kainate-evoked currents and 44% of 10 μM kainate-evoked currents, indicating that, at these concentrations, both AMPAR and KAR are activated. GYKI only reduced 3 μM kainate-evoked currents by 2%, indicating that KARs are selectively activated at this concentration. *n* = 3 cells from 1 mouse at P14, P15, or P22 for each kainate concentration. (D) Applying the KAR-specific agonist SYM2081 (SYM) did not evoke any currents in OPCs, in contrast to 3 μM kainate. SYM, *n* = 8 cells from 3 P13-15 or P40-58 mice; kainate, *n* = 11 cells from 5 P13-15 or P20-36 mice. (E) Hippocampal neurons had similar densities of 10 μM SYM- and 3 μM kainate-evoked currents. SYM, *n* = 8 cells from 4 coverslips; kainate, *n* = 5 cells from 1 coverslip. (F) The 30 μM kainate-evoked currents recorded in OPCs after an 8-min incubation with control artificial cerebrospinal fluid (aCSF) or 0.3 mg/mL Concanavalin A (Con A), a blocker of KAR desensitization. Incubation with Con A did not potentiate 30 μM kainate-evoked currents in OPCs. Control, *n* = 5 cells from 2 P20-36 mice; Con A, *n* = 10 cells from 6 P13-15, P40-58, P182 mice. (G) The 30 μM kainate-evoked currents recorded in hippocampal neurons before and after an 8-min incubation with 0.3 mg/mL Con A. Incubation with Con A did not alter 30 μM kainate-evoked currents in hippocampal neurons. *n* = 3 cells from 3 coverslips. (H) The 3 μM kainate-evoked currents recorded in OPCs after a 15-min incubation with control aCSF or 50 μM UBP301, a KAR-selective blocker. UBP301 did not block 3 μM kainate-evoked currents in OPCs beyond normal current rundown. *n* = 3 cells from 1 P20-36 or P99 mouse for each condition. (I) The 3 μM kainate-evoked currents recorded in hippocampal neurons before and after a 15-min incubation with 50 μM UBP301. UBP301, 50 μM, partially blocked 3 μM kainate-evoked currents in hippocampal neurons. *n* = 6 cells from 6 coverslips. Data are shown as mean ± SEM, with gray dots indicating individual recorded cells. Statistics were calculated with one-sample t tests (C, G, I) or unpaired two-tailed t tests (D, E, F, H).

First, to validate that OPCs express functional KARs *in vivo*, we identified a kainate concentration that would selectively activate KARs. To do so, we bath-applied 10 μM GYKI53655 (GYKI), an AMPAR-specific antagonist,^37^ and 30, 10, or 3 μM kainate on voltage-clamped cortical NG2-EYFP^+^ OPCs^34^ in acute brain slices (Figures 1A and 1B). We found that GYKI blocked 66% of the 30 μM kainate-evoked steady-state currents and 44% of the 10 μM kainate-evoked steady-state currents (*p* = 0.008, *p* = 0.09, respectively; Figures 1B and 1C), indicating that, at these concentrations, kainate activates both AMPARs and KARs in OPCs. In contrast, GYKI had no effect on 3 μM kainate-evoked steady-state currents (*p* = 0.2; Figures 1B and 1C). This suggests that 3 μM kainate specifically activates KARs in OPCs, in line with dose-response curves established in hippocampal neurons.^38^ Thus, OPCs have functional KARs, which can be pharmacologically isolated with low kainate concentrations.

### OPC KARs do not respond to neuronal KAR-specific drugs

Little is known about KARs in OPCs, and it is unclear how similar these receptors are to neuronal KARs. KARs can be formed of five subunits, GluK1–5. GluK1–3 can assemble into functional homomeric receptors, while GluK4–5 must assemble with at least one of GluK1–3 to form conducting receptors.^39^^,^^40^ In neurons, KARs are heterogeneous; for instance, dorsal root ganglia (DRG) neurons mostly express GluK1,^41^^,^^42^ whereas hippocampal neurons express all five subunits.^43^ OPCs have transcripts for all five *Grik* subunits^20^^,^^44^^,^^45^^,^^46^^,^^47^ (Figure S1), but a previous pharmacological study suggests that OPCs lack GluK1,^30^ while western blots of cultured OPCs suggest protein expression of GluK2/3 and GluK5,^31^ although the presence of GluK1 and GluK4 was not tested.

To investigate functional KAR subunit membrane surface expression in OPCs *in vivo*, we took advantage of KAR-specific drugs developed on recombinant receptors or DRG neurons. Importantly, these drugs have different effects on DRG and hippocampal neurons, indicating that they may have subunit specificity.^41^^,^^42^^,^^48^ Thus, we examined whether KARs in OPCs are similar to those in DRG neurons or hippocampal neurons.

We first tested whether SYM2081, a KAR-specific agonist,^49^ elicited currents in cortical OPCs. SYM2081 evokes currents in recombinant GluK1, GluK2, or GluK3 receptors, and in both DRG and hippocampal neurons,^33^^,^^50^^,^^51^^,^^52^^,^^53^^,^^54^ but binds GluK1 with highest affinity.^55^^,^^56^^,^^57^ We did not detect any 10 μM SYM2081-evoked responses in cortical OPCs (Figure 1D). In contrast, in cultured hippocampal neurons, 10 μM SYM2081 evoked currents of a density similar to that of 3 μM kainate (*p* = 0.6; Figure 1E). These data suggest that OPC KARs may not be activated by SYM2081 and therefore that GluK1 may not be a main contributor to OPC KAR currents, in line with OPCs not responding to ATPA, a GluK1-specific agonist,^30^ and low *Grik1* transcript levels (Figure S1).^20^

As SYM2081 did not elicit any currents in OPCs, we tested Concanavalin A (Con A), a lectin that selectively blocks KAR desensitization and therefore potentiates KAR steady-state currents. Previous studies indicate that Con A potentiates GluK1, GluK2, GluK1/4, and GluK2/4 recombinant receptor currents, as well as native DRG KAR currents,^42^^,^^58^ but shows little potentiation of recombinant GluK3 receptor currents or hippocampal neuron KAR currents (*p* = 0.6; Figure 1G)^48^^,^^50^^,^^58^ and does not act on AMPARs.^42^ Kainate-evoked currents run down with time in OPCs (Figure S2), and we therefore compared the change in kainate-evoked currents before and after an 8 min application of Con A^42^^,^^48^^,^^58^ or control recording solution. Con A-treated OPCs did not differ from control OPCs (*p* = 0.6; Figure 1F), suggesting that, like SYM2081, Con A has little detectable effect on OPC KARs. These data suggest that neither GluK1 nor GluK2 contributes significantly to KAR currents in OPCs.

Finally, we also tested whether 50 μM UBP301, a KAR-specific antagonist, blocked cortical OPC KAR currents. To date, UBP301 has only been tested on the dorsal root,^59^ where neurons mostly express GluK1,^41^^,^^42^ or on recombinant GluK3 receptors, albeit at a high concentration that also antagonizes AMPAR.^59^^,^^60^ Moreover, crystallography data indicate that UBP301 only partially binds GluK3, keeping the channel in a closed state differing from GluK1 where the channel is completely closed.^60^ Hence it is still unclear to what extent 50 μM UBP301 would reduce current conduction of GluK3-containing receptors. Although UBP301 partially reduced KAR currents in hippocampal neurons (*p* = 0.02, Figure 1I), it did not have any detectable effect on OPCs (*p* = 0.3; Figure 1H). Collectively, our data suggest that KAR-specific drugs that were mostly developed on GluK1-containing DRG neurons have little effect on OPC KAR, suggesting that OPC KAR may differ from both DRG and hippocampal neurons.

To further investigate OPC KAR subunit composition, we reanalyzed existing bulk RNA sequencing data and found that, although OPCs have transcripts for all five subunits, they have the lowest levels of *Grik1* and *Grik4* transcripts (Figure S1). As transcripts do not always correspond to protein levels,^20^ we enriched forebrain samples for OPCs with magnetic activated cell sorting and performed western blots. We detected bands at the expected molecular weight for GluK2, GluK3, GluK4, and GluK5, but no clear band for GluK1 (Figure S3), consistent with previous *in vitro* or pharmacological work^30^^,^^31^ and our pharmacological electrophysiological data.

We recorded whole-cell currents and therefore the sum of all receptor subunit combinations present in single OPCs. However, our electrophysiological, transcriptomic, and western blot data allow us to infer potential KAR subunit composition in OPCs. *Grik2*, *Grik3*, and *Grik5* transcript levels were higher than those of other subunits, suggesting that they could be the main subunits present in OPCs. However, GluK5 and GluK4 conduct current only when assembled with GluK1, GluK2, or GluK3. Collectively, our electrophysiological, transcriptomic, and western blot data suggest that OPCs lack GluK1, and thus, OPCs may predominantly express receptor combinations that could include GluK2/5, GluK3/5, GluK2/4, or GluK3/4. The inclusion of GluK4 and GluK5 would alter agonist and antagonist responses, though the effects of GluK4-containing receptors remain less well characterized. This is consistent with GluK5 altering the agonist efficacy of SYM2081 and, for example, reducing SYM2081 potency when assembled with GluK2.^53^ Con A is known to potentiate GluK2-containing receptors but has little effect on GluK3-containing receptors.^48^ Thus, the lack of detectable potentiation by Con A raises the possibility that GluK2/5 or GluK2/4 receptors are not the main contributor to KAR currents in OPCs, but rather that GluK3/5 and GluK3/4 might be. Importantly, this indicates that the existing KAR pharmacology does not allow for the isolation of KAR currents in OPCs as subunit selective agonists have been developed for GluK1 only and that the application of 3 μM kainate remains the most suitable approach to study KAR function in OPCs.

### AMPA receptor current density changes with age in OPCs

**Figure 2:**
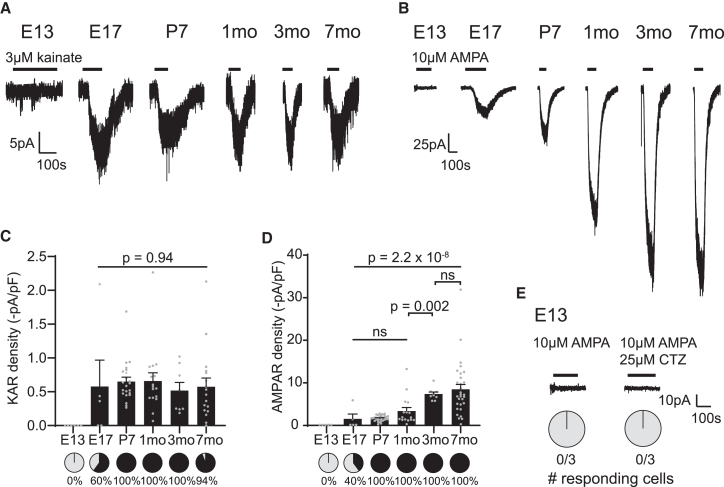
AMPAR density increases with age in OPCs (A) Representative 3 μM kainate-evoked currents recorded in OPCs between E13 and 7 months (mo). Embryonic currents were recorded in the forebrain, and the postnatal currents shown here were recorded in the cortex. (B) The 10 μM AMPA-evoked currents recorded in OPCs between E13 and 7 months. Embryonic currents were recorded in the forebrain, and the postnatal currents shown here were recorded in the cortex. (C) KARs were first detected at E17, with the proportion of responding cells (pie charts) increasing between embryonic and postnatal OPCs (*p* = 6.9x10^−8^, χ^2^), while KAR density (bar graph) remained stable between E17 and 7 months. E13, *n* = 6 cells from 3 litters; E17, *n* = 5 cells from 1 litter; P7, *n* = 22 cells from 5 mice; 1 month, *n* = 16 cells from 4 mice; 3 months, *n* = 8 cells from 4 mice; 7 months, *n* = 17 cells from 8 mice. Cortical and callosal data are pooled within postnatal timepoints. (D) AMPARs were first detected at E17, and the proportion of cells with AMPA-evoked currents increased between embryonic and postnatal OPCs (p < 1x10^−15^, χ^2^). AMPAR density increased with age. E13, *n* = 5 cells from 3 litters; E17, *n* = 5 cells from 1 litter; P7, *n* = 32 cells from 7 mice; 1 month, *n* = 16 cells from 4 mice; 3 months, *n* = 9 cells from 4 mice; 7 months, *n* = 30 cells from 9 mice. Cortical and callosal data are pooled within postnatal timepoints. (E) AMPA, 10 μM, did not evoke currents in E13 OPCs, even in the presence of 25 μM cyclothiazide (CTZ), a blocker of AMPAR desensitization. *n* = 3 cells from 3 litters. The AMPA-only recording on the left is the same as in B. Data are shown as mean ± SEM, with gray dots indicating individual recorded cells. Statistics were calculated with ANOVA (*p*-values above) followed by Holm-Bonferroni post hoc tests (*p*-values below).

We next sought to examine how KAR and AMPAR steady-state current densities change with age. To establish this, we used carefully selected concentrations that ensure specificity of the agonist for each receptor: 3 μM kainate (Figures 1B and 1C) and 10 μM AMPA.^61^^,^^62^ We bath-applied either agonist to NG2-EYFP^+^ OPCs from two embryonic timepoints: E13, when forebrain OPCs are first established but lack 30 μM kainate-evoked responses,^20^^,^^63^ and E17; and to OPCs in the cortex and corpus callosum in four postnatal timepoints: P7, 1 month, 3 months, and 7 months. At E13, neither AMPA nor kainate evoked currents in OPCs (Figures 2A–2D), in line with a previous report indicating that E13 OPCs do not respond to 30 μM kainate.^20^ By E17, both AMPA- and kainate-evoked currents were detected (in 40% and 60% cells, respectively). Postnatally, all cortical and callosal OPCs had detectable AMPA-evoked responses, and AMPA-evoked current density increased with age (*p* = 2.2x10^−8^; Figures 2B and 2D). In contrast, while most postnatal cortical and callosal OPCs had detectable kainate-evoked currents, current density remained stable from E17 onward (*p* = 0.94; Figures 2A and 2C).

To further validate that 3 μM kainate selectively activates KARs and not AMPARs, we plotted 10 μM AMPA-evoked currents with age in OPCs. AMPA-evoked currents increased with age (*p* = 9.1x10^−5^; Figure S4A), and thus, if 3 μM kainate activated AMPARs, we would expect to see a similar fold increase in current size with age (Figure S4B). However, 3 μM kainate-evoked currents decreased with age in OPCs, although current density remained stable (*p* = 0.003, Figures S4 and 2C). This suggests that OPCs express functional KARs throughout life and that KAR steady-state current density remains stable with age.

As both KARs and AMPARs can desensitize, we tested whether the lack of detectable AMPA-evoked response in the early embryo indicated the absence of functional receptors or rapid desensitization of steady-state currents. We bath-applied AMPA in the presence of cyclothiazide (CTZ), an AMPAR desensitization blocker,^42^ in E13 OPCs. We did not detect any AMPA-evoked currents in the presence of CTZ (Figure 2E), suggesting that the absence of detectable currents indicates the absence of functional AMPAR. Collectively, this indicates that AMPARs appear in OPCs by E17, and that with age, OPCs gradually acquire larger AMPAR densities.

These data show that, in OPCs, KAR current density remains stable throughout life, whereas AMPAR current density changes with age, driving the age-related increase in AMPAR/KAR current density previously identified.^20^

### AMPAR current density increases with age at different rates between regions

**Figure 3:**
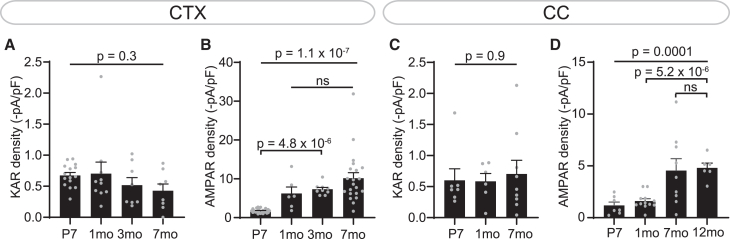
AMPAR density increases at different rates between regions (A) KAR density did not differ with age in the cortex (CTX). P7, *n* = 15 cells from 5 mice; 1 month, *n* = 10 cells from 4 mice; 3 months, *n* = 8 cells from 4 mice; 7 months, *n* = 8 cells from 3 mice. (B) In the cortex, AMPAR density increased between P7 and 1 month (mo) and remained stable afterward. P7, *n* = 25 cells from 7 mice; 1 month, *n* = 6 cells from 3 mice; 3 months, *n* = 9 cells from 4 mice; 7 months, *n* = 21 cells from 9 mice. (C) KAR density did not differ with age in the corpus callosum (CC). P7, *n* = 7 cells from 3 mice; 1 month, *n* = 6 cells from 3 mice; 7 months, *n* = 9 cells from 6 mice. (D) In the corpus callosum, AMPAR density did not increase until 7 months and remained stable afterward. P7, *n* = 7 cells from 4 mice; 1 month, *n* = 12 cells from 5 mice; 7 months, *n* = 9 cells from 6 mice; 12 months, *n* = 6 cells from 3 mice. Data are shown as mean ± SEM, with gray dots indicating individual recorded cells. Statistics were calculated by ANOVA (*p*-values above) and Holm-Bonferroni post hoc tests (*p*-values below).

AMPAR/KAR current density differs temporally and regionally,^20^ and as AMPAR but not KAR current density differed with age, we investigated whether AMPARs, KARs, or both differ between gray and white matter. KAR current density did not differ with age in either the cortex (*p* = 0.3) or corpus callosum (*p* = 0.9), nor did it differ between both regions (Figures 3A and 3C). In contrast, AMPAR current density increased at different rates between the cortex and corpus callosum. In the cortex, AMPAR current density increased after the first postnatal week and remained stable afterward (*p* = 4.8x10^−4^; Figure 3B). In the corpus callosum, AMPAR density was lower than in the cortex (Figure S5) and only increased by 7 months, remaining stable afterward (*p* = 5.2x10^−6^; Figure 3D). We detected a small difference in AMPAR current density between gray and white matter OPCs during the first postnatal week, which became more pronounced in >1-month-old mice (Figure S5). Thus, our data suggest that, unlike other ion channels in OPCs,^20^ KAR functional membrane surface expression is regionally and temporally homogeneous, while AMPARs differ between gray and white matter.

### AMPAR Ca^2+^ permeability differs with age and between regions

We next asked whether these temporal and regional differences in AMPAR current density were driven by a difference in the number of receptors or by differences in subunit composition resulting in larger single-channel conductance. AMPARs can be formed of four subunits, GluA1–4, and we find that all AMPAR transcripts are expressed in OPCs and seem to vary with cell state (Figure S1). In postnatal tissues, the GluA2 subunit is edited at the Q/R site, making it impermeable to Ca^2+^.^35^^,^^64^ As Ca^2+^-permeable AMPARs have higher single-channel conductance than GluA2-containing Ca^2+^-impermeable AMPARs^65^ and two studies suggest that AMPAR Ca^2+^ permeability differs with age in callosal and hippocampal OPCs,^66^^,^^67^ we asked if an increase in Ca^2+^ permeability could underlie the increase in AMPAR current density in OPCs with age.

**Figure 4:**
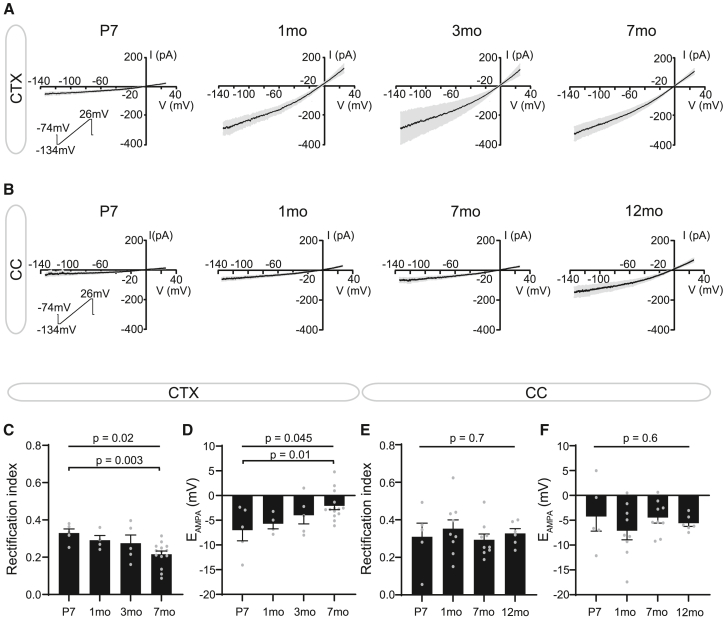
Ca^2+^ permeability increases with age in cortical OPCs (A) Mean current voltage relationship (inset, voltage ramp −134 mV–26 mV) for 10 μM AMPA-evoked currents in cortical OPCs (CTX) across different timepoints. (B) Mean current voltage relationship (inset, voltage ramp −134 mV–26 mV) for 10 μM AMPA-evoked currents in callosal OPCs (CC) across different timepoints. (C) The rectification index decreased with age in cortical OPCs. (D) In the cortex, the AMPA reversal potential (E_AMPA_) approached 0 mV with age. (E) The rectification index did not differ with age in callosal OPCs. (F) In the corpus callosum, E_AMPA_ did not differ with age. Data are shown as mean ± SEM, with gray dots indicating individual recorded cells. CTX: P7, *n* = 5 cells from 3 mice; 1 month, *n* = 4 cells from 3 mice; 3 months, *n* = 5 cells from 2 mice; 7 months, *n* = 14 cells from 6 mice. CC: P7, *n* = 5 cells from 3 mice; 1 month, *n* = 9 cells from 5 mice; 7 months, *n* = 9 cells from 6 mice; 12 months, *n* = 6 cells from 3 mice. Statistics were calculated by ANOVA (*p*-values above) and Holm-Bonferroni post hoc tests (*p*-values below).

First, we examined whether AMPAR Ca^2+^ permeability differed with age in the cortex and corpus callosum. To address this, we applied voltage ramps at the peak of the AMPA-evoked response (Figures 4A and 4B) and calculated the rectification index (RI, see STAR Methods) and reversal potential (E_AMPA_), two indirect measures of AMPAR Ca^2+^ permeability. The RI calculated from these voltage ramps ranged between 0.06 and 0.49, suggesting a mixed population of both Ca^2+^-permeable and impermeable AMPARs, in line with previous reports.^27^^,^^28^^,^^29^^,^^66^^,^^67^^,^^68^^,^^69^^,^^70^^,^^71^ In the cortex, the RI gradually decreased with age (*p* = 0.02) while E_AMPA_ gradually became less negative with age (*p* = 0.045; Figures 4C and 4D), suggesting a higher contribution of ions with positive equilibrium potentials such as Ca^2+^. Taken together, these data indicate an increase in AMPAR Ca^2+^ permeability with age in cortical OPCs.^35^^,^^72^^,^^73^^,^^74^^,^^75^ In contrast, the RI and E_AMPA_ remained constant in the corpus callosum (Figures 4E and 4F). Although the RI and E_AMPA_ did not differ between cortical and callosal OPCs in the first postnatal week (*p* = 0.8, *p* = 0.5, respectively), the RI was higher (*p* = 0.03) and E_AMPA_ more negative (*p* = 0.04) in the adult corpus callosum than in the adult cortex (Figures S5C–S5F). This suggests that Ca^2+^ permeability is lower in the adult corpus callosum compared with that in the adult cortex, and remains stable with age. Contrary to our findings, a previous report suggests that Ca^2+^ permeability increases in the corpus callosum between P7-8 and P42-52.^66^ A potential explanation of this divergence is that we did not include spermine in our intracellular solution and therefore small differences in RI in the corpus callosum may not have been detectable; however, this suggests that the increase in Ca^2+^ permeability in cortical OPCs may in fact be more pronounced than we report.

Of note, in contrast to previous reports analyzing Ca^2+^ permeability on evoked excitatory postsynaptic potentials (EPSCs),^66^^,^^69^ we bath-applied AMPA. Thus, we may have recorded both synaptic and extra-synaptic AMPAR currents. However, a number of studies suggest that OPC AMPARs are located at the neuron-OPC synapse, as the timescale of AMPAR activation is similar to that of neuronal synapses, and suggests localization facing the presynaptic release sites.^76^ In addition, the RI we measured on bath-application-evoked currents in callosal OPCs at P7 (0.31 ± 0.07; *n* = 5) did not differ from the one measured on evoked EPSCs in P12-17 callosal OPCs (0.33 ± 0.06; *n* = 6; *p* = 0.8, unpaired two-tailed t test),^69^ indicating that we are likely recording currents from similar populations of receptors.

Taken together, these data indicate that with age there is a gradual accumulation of cortical OPCs with higher AMPAR Ca^2+^ permeability. This gradual change contrasts with the early increase in AMPAR current density at 1 month (Figure 3B), suggesting that Ca^2+^ permeability only partially underlies the age-driven increase in AMPAR current density in cortical OPCs.

### Flip and flop AMPAR variants do not change with age in OPCs

As AMPAR current density rapidly increased at 1 month in cortical OPCs and by 7 months in callosal OPCs while Ca^2+^ permeability gradually increased with age in the cortex and did not change in the corpus callosum, we asked if AMPARs in OPCs undergo further changes with age. All four AMPAR subunits can be alternatively spliced, resulting in two possible variants, flip and flop, which exhibit different current responses and desensitization^36^ and have different expression patterns with age in neurons.^77^ Hence, we asked if the temporal increase in AMPAR density in OPCs could be explained by a different ratio of flip and flop variants.

To examine this, we tested whether potentiation of AMPA-evoked steady-state currents by PEPA (2-[2,6-Difluoro-4-[[2-[(phenylsulfonyl)amino]ethyl]thio]phenoxy]acetamide) and CTZ, two AMPAR allosteric modulators that block desensitization, changed with age in the cortex and corpus callosum. While PEPA preferentially acts on flop subunits, CTZ preferentially acts on flip subunits, and therefore, measuring their effect on AMPAR currents provides an indication of the relative abundance of flip and flop variants.^78^^,^^79^^,^^80^

**Figure 5:**
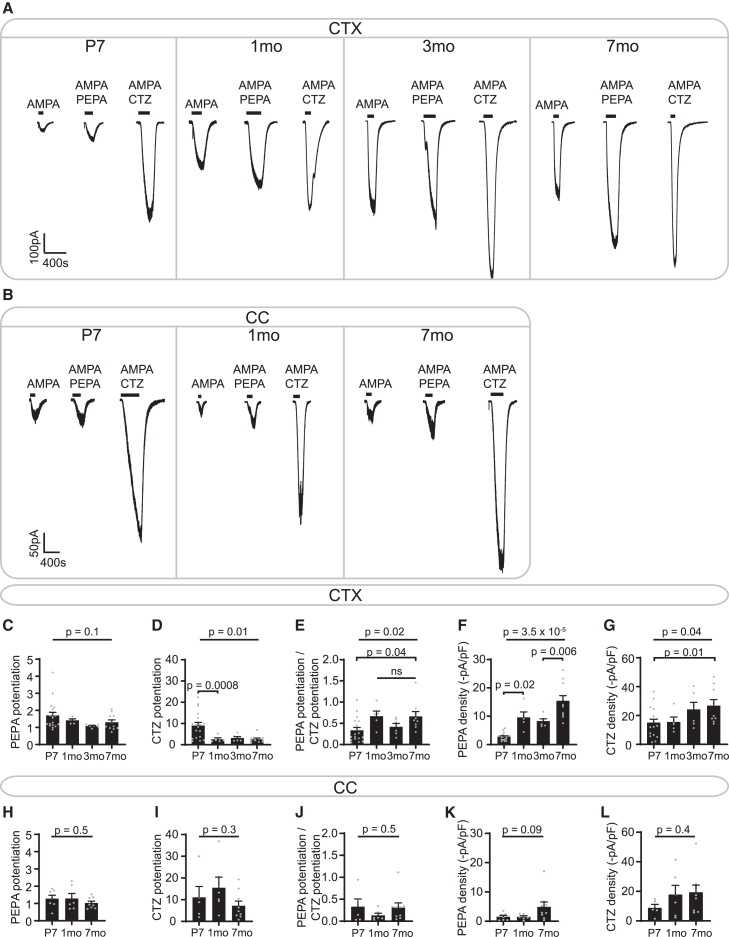
AMPAR desensitization decreases with age in cortical OPCs (A) Currents evoked by 10 μM AMPA, 10 μM AMPA and 25 μM PEPA, and 10 μM AMPA and 25 μM CTZ in cortical OPCs (CTX) at different ages. (B) Currents evoked by 10 μM AMPA, 10 μM AMPA and 25 μM PEPA, and 10 μM AMPA and 25 μM CTZ in callosal OPCs (CC) at different ages. (C) Potentiation of AMPA-evoked currents by PEPA did not differ with age in cortical OPCs. P7, *n* = 20 cells from 5 mice; 1 month, *n* = 5 cells from 3 mice; 3 months, *n* = 6 cells from 2 mice; 7 months, *n* = 11 cells from 7 mice. (D) Potentiation of AMPA-evoked currents by CTZ decreased between P7 and 1 month (mo) in cortical OPCs. P7, *n* = 18 cells from 5 mice; 1 month, *n* = 5 cells from 3 mice; 3 months, *n* = 6 cells from 2 mice; 7 months, *n* = 9 cells from 6 mice. (E) The ratio between PEPA potentiation and CTZ potentiation of AMPA-evoked currents increased between P7 and 1 month in cortical OPCs and remained stable afterward. P7, *n* = 18 cells from 5 mice; 1 month, *n* = 5 cells from 3 mice; 3 months, *n* = 6 cells from 2 mice; 7 months, *n* = 9 cells from 6 mice. (F) AMPA, 10 μM, and 25 μM PEPA-evoked current density increased with age in cortical OPCs. P7, *n* = 20 cells from 5 mice; 1 month, *n* = 5 cells from 3 mice; 3 months, *n* = 6 cells from 2 mice; 7 months, *n* = 11 cells from 7 mice. (G) AMPA, 10 μM, and 25 μM CTZ-evoked current density increased with age in cortical OPCs P7, *n* = 18 cells from 5 mice; 1 month, *n* = 5 cells from 3 mice; 3 months, *n* = 6 cells from 2 mice; 7 months, *n* = 9 cells from 6 mice. (H) PEPA potentiation of AMPA-evoked currents did not differ with age in callosal OPCs. P7, *n* = 7 cells from 3 mice; 1 month, *n* = 6 cells from 3 mice; 7 months, *n* = 9 cells from 6 mice. (I) CTZ potentiation of AMPA-evoked currents did not differ with age in callosal OPCs. P7, *n* = 5 cells from 3 mice; 1 month, *n* = 6 cells from 3 mice; 7 months, *n* = 9 cells from 6 mice. (J) The ratio between PEPA potentiation and CTZ potentiation of AMPA-evoked currents did not differ with age in callosal OPCs. P7, *n* = 5 cells from 3 mice; 1 month, *n* = 6 cells from 3 mice; 7 months, *n* = 9 cells from 6 mice. (K) AMPA, 10 μM, and 25 μM PEPA-evoked current density did not differ with age in callosal OPCs. P7, *n* = 7 cells from 3 mice; 1 month, *n* = 6 cells from 3 mice; 7 months, *n* = 9 cells from 6 mice. (L) AMPA, 10 μM, and 25 μM CTZ-evoked current density did not differ with age in callosal OPCs. P7, *n* = 5 cells from 3 mice; 1 month, *n* = 6 cells from 3 mice; 7 months, *n* = 9 cells from 6 mice. Data are shown as mean ± SEM, with gray dots indicating individual recorded cells. Statistics were calculated by ANOVA (*p*-values above) and Holm-Bonferroni post hoc tests (*p*-values below).

In the corpus callosum, neither PEPA (flop) nor CTZ (flip) potentiation changed with age (*p* = 0.5, *p* = 0.3, respectively), and the PEPA to CTZ potentiation ratio therefore remained stable with age (*p* = 0.5; Figures 5B, 5H–5L). This ratio ranged between 0.03 and 0.49 for most cells, suggesting that AMPAR in callosal OPCs are composed mostly of flip subunits.^80^ Of note, these ratios were previously determined on recombinant receptors using 100 μM PEPA and CTZ; however, we found that potentiation by 25 μM PEPA and CTZ did not differ from potentiation by 100 μM PEPA and CTZ (*p* = 0.2, *p* = 0.05, respectively; Figures S6G and S6H), and therefore, our measured ratios are comparable with previously reported ratios. Taken together, our data suggest that the relative abundance of flip and flop variants does not differ with age in the white matter.

In the cortex, PEPA (flop) potentiation of AMPAR steady-state currents remained stable with age, as in the white matter (*p* = 0.1). However, the potentiation by CTZ (flip) decreased between P7 and 1 month (*p* = 0.0008; Figures 5A–5C, 5D). Although the PEPA to CTZ ratio increased with age (*p* = 0.02; Figure 5E), PEPA potentiation of AMPAR steady-state currents was unaltered, indicating that an increase in the relative abundance of flop variants does not drive this change. Moreover, the ratio of PEPA to CTZ potentiation ranged between 0.04 and 1.4, suggesting that cortical OPCs express a mix of receptors composed of either mostly flip variants or both flip and flop variants, but no receptors composed of flop variants only, as these would have a PEPA to CTZ potentiation ratio above 1.86.^80^ Collectively, these results suggest that OPCs mostly express flip subunits and that changes in the abundance of flip and flop variants are unlikely to underlie the increase in AMPAR current density. Nevertheless, CTZ potentiation of AMPAR currents declines with age in cortical OPCs, indicating a decrease in CTZ efficacy with age.

### AMPAR desensitization differs with age and between regions

A decrease in CTZ efficacy could reflect a decrease in AMPAR desensitization, as CTZ acts as a desensitization blocker. Reduced desensitization could in turn result in the larger steady-state AMPAR currents that we observed with age (Figure 3B). If a decrease in AMPAR desensitization drives the decline in CTZ potentiation, we would expect the AMPA+CTZ current density to remain constant even if CTZ potentiation decreases. In the cortex, AMPA+CTZ current density only increased at 7 months (*p* = 0.01; Figure 5G), coinciding with the increase in Ca^2+^ permeability but not with the increase in AMPAR current density or the decrease in CTZ potentiation that occurred at 1 month. In contrast, the AMPA+PEPA current density tightly followed the increase in AMPAR density at 1 month (*p* = 0.02; Figure 5F), consistent with PEPA having little effect on OPC AMPAR currents. This indicates that the decrease in CTZ efficacy in 1 month OPCs is mediated by a decrease in AMPAR desensitization, which likely underlies the increase in AMPAR current density.

As CTZ potentiation decreased in the cortex but not in the corpus callosum, and AMPAR current density is larger in the cortex, we asked whether AMPAR desensitization might differ between both regions. At P7, CTZ potentiation did not differ between cortical and callosal OPCs (*p* = 0.6; Figure S6C). However, after 1 month, CTZ potentiation was higher in the corpus callosum (*p* = 0.007; Figure S6D), suggesting that callosal OPCs desensitize more than cortical OPCs. In addition, we found that the variance in CTZ potentiation was lower in the cortex after 1 month compared with the neonatal cortex or the corpus callosum at all timepoints (Figures S6E and S6F), suggesting that AMPAR receptors may be more homogeneous in >1-month-old cortical OPCs.

**Figure 6:**
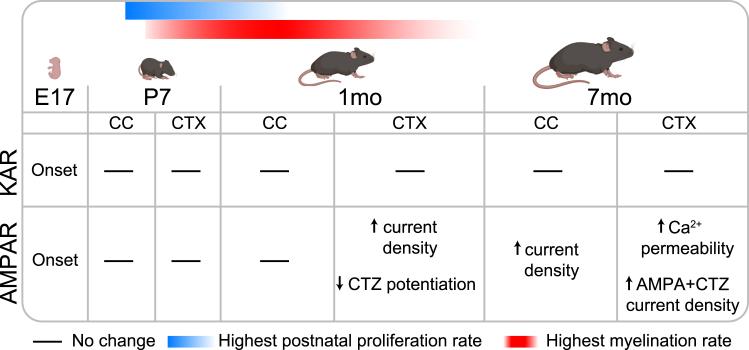
Summary of the temporal and regional changes in OPC AMPARs and KARs Timing of the appearance of KARs and AMPARs and changes in AMPAR properties, alongside major postnatal proliferation^20^ and myelination^81^-related milestones. Mouse schematics created with BioRender.com.

Collectively, our data indicate that, by the first postnatal month, cortical OPCs acquire larger AMPAR current densities resulting first from a decrease in AMPAR desensitization and later from an increase in Ca^2+^ permeability. In contrast, callosal OPCs do not display any marked age-driven changes in AMPAR desensitization or Ca^2+^ permeability and only acquire larger AMPAR current densities by 7 months (Figure 6). Remarkably, these temporal and regional changes coincide with differences in proliferation and differentiation potential between gray and white matter OPCs,^1^^,^^20^^,^^82^ raising the possibility that the kinetics of AMPAR activation in OPCs may contribute to modulate cell fate.

## Discussion

We sought to determine whether AMPARs, KARs, or both differ with age and between regions. We identified that OPCs first appear without AMPARs or KARs, acquire AMPARs and KARs by E17, and have functional AMPARs and KARs in the cortex and corpus callosum until at least 7 months of age. We show that AMPAR current density increases with age in the cortex, driven by reduced desensitization and increased Ca^2+^ permeability, while remarkably, KAR current density does not differ with age, or between gray and white matter, in contrast to other glutamate receptors or ion channels,^20^ suggesting that KARs may perhaps play an essential role in OPCs.

Although the function of KAR signaling in OPCs is currently unknown, activating KARs in subventricular zone neural progenitors promotes their survival and proliferation in culture, while blocking KARs in subventricular zone neuroblasts increases their migration *ex vivo.*^83^^,^^84^ Conceivably, KAR signaling may play a similar role in OPCs, with constant low-level signaling promoting survival, migration, and basal proliferation levels. However, KAR pharmacology is limited, and we found that the KAR-specific drugs SYM2081, Con A, and UBP301 had little effect on OPCs, preventing further studies of KAR function. Nonetheless, these data combined with transcriptomic and western blot data suggest that OPCs might have a distinct subunit composition, lacking GluK1 and mostly composed of GluK3/5 and GluK3/4, with some small contribution of GluK2/5 and GluK2/4, or even tetramers incorporating all four subunits. Further characterizing KAR composition and function in OPCs may provide new specific therapeutic targets, as OPCs may have unique KARs, and especially as OPC KAR current density remains stable with age and between regions, suggesting that KAR-specific interventions might act on the majority of OPCs throughout life.

In contrast to KAR current density, AMPAR current density increased with age in postnatal OPCs, underlying the increase in 30 μM kainate-evoked current density we reported previously.^20^ Our data suggest that this increase in AMPAR current density results from a decrease in AMPAR desensitization rather than from changes in the relative abundance of flip/flop subunits, as PEPA potentiation was stable with age. Reduced desensitization could be driven by further RNA editing at the R/G site^85^^,^^86^; a change in subunit composition as, for example, GluA3 desensitizes less than GluA1^87^ and therefore exhibits weaker potentiation by CTZ^80^; or by auxiliary subunits.^88^ For instance, auxiliary subunits can alter receptor desensitization and potentiation by CTZ^88^ or dampen the polyamine block of Ca^2+^-permeable AMPAR^89^ and have been shown to regulate AMPAR subunit composition in an OPC line.^75^ However, reanalysis of our previously published transcriptional dataset^20^ implies no postnatal changes in AMPAR subunit or auxiliary subunit transcript levels (Figure S1), implying that changes in AMPAR subunit composition or auxiliary subunits may not underlie reduced desensitization. Nevertheless, transcript levels may not necessarily relate to protein levels in OPCs,^20^ warranting further studies into changes in AMPAR subunit composition and auxiliary AMPAR subunits functional expression in OPCs with age.

Further examining AMPAR properties in OPCs, we found that Ca^2+^ permeability gradually increased with age in the cortex, but not in the corpus callosum, likely contributing to the cortical increase in AMPAR current density. Surprisingly, constitutively knocking out the GluA2 subunit in oligodendrocyte lineage cells does not alter kainate-evoked current density in P14 callosal OPCs despite increasing Ca^2+^ permeability, suggesting that Ca^2+^ permeability may have a limited effect on AMPAR current density in OPCs, although the authors suggest there may have been compensation.^28^ While this constitutive GluA2 knockout does not alter OPC proliferation or the density of oligodendrocyte lineage cells at P14,^28^ altering AMPAR Ca^2+^ permeability postnatally has been shown to modulate OPC proliferation and differentiation.^69^^,^^90^ For instance, increasing Ca^2+^ permeability in the corpus callosum between P10 and P17 promotes proliferation and blocks differentiation.^69^ In contrast, decreasing Ca^2+^ permeability in neonatal mice by overexpressing GluA2 has no effect, but overexpressing GluA2 in adult mice increases OPC proliferation.^90^ This is consistent with the gradual accumulation of Ca^2+^-permeable AMPAR with age that we report here and implies that this accumulation may contribute to the decrease in proliferation rate^1^^,^^20^ and the failure in remyelination^91^ with age, especially as overexpressing GluA2 promotes both proliferation and differentiation in lysolecithin lesions.^90^

Intriguingly, Ca^2+^-permeable AMPARs are upregulated in GluN1 knockout OPCs, which do not have functional NMDARs, suggesting that there may be a negative correlation between NMDAR and Ca^2+^-permeable AMPAR membrane expression.^92^ We recently proposed that loss of NMDAR in aged mice might coincide with increased AMPAR/KAR current density, and entry into a quiescent state, with limited proliferation and differentiation potential.^20^^,^^93^ In line with our work, reanalysis of a single-cell sequencing dataset^47^ indicates that *Gria2* transcripts are lower in quiescent OPCs compared with those in cycling OPCs (Figure S1), suggesting increased Ca^2+^ permeability in quiescent OPCs. Nonetheless, single-cell sequencing cannot account for post-translation modifications, which are known to regulate neurotransmitter receptor trafficking and membrane insertion.^94^ Considering our findings that AMPAR Ca^2+^ permeability increases with age in the cortex, but not in the corpus callosum, where OPCs proliferate and differentiate at a higher rate^1^ and maintain NMDARs later,^20^ it is possible that quiescent OPCs might be defined by loss of NMDAR and upregulation of Ca^2+^-permeable AMPAR. Consistent with this, decreasing Ca^2+^ permeability in adult OPCs promotes proliferation,^90^ and a recent report in zebrafish found that quiescent OPCs residing in neuronal-rich areas, which do not differentiate into mature oligodendrocytes, exhibit more calcium transients than OPCs residing in axonal-rich areas,^95^ perhaps reflecting the increase in AMPAR Ca^2+^ permeability we detected in gray matter but not in white matter.

In summary, we show that KAR expression is stable in OPCs until at least 7 months of age. This raises the possibility that KAR signaling may play a role in survival and/or proliferation, and perhaps provide a unique target to manipulate OPCs’ sensitivity to neuronal activity, although further studies are needed to parse out the role of KARs in OPCs. In contrast, AMPAR properties differ with age and region, and our findings provide an initial roadmap of these changes. Given the conflicting evidence for the role of AMPAR signaling in regulating OPC fate and myelination,^27^^,^^28^^,^^69^^,^^90^ it is becoming clear that understanding the subunit composition and properties of OPC AMPARs is critical to develop OPC-specific drugs to target AMPAR signaling and, for instance, promote myelin regeneration.

### Limitations of the study

Here, we used whole-cell patch-clamp to investigate regional and temporal diversity in OPC AMPARs and KARs. We found that KAR current density remained stable with age but that neuronal KAR-specific drugs had little effect on OPCs, likely due to a different subunit composition. The lack of KAR pharmacology that specifically acts on OPCs’ KAR subunit combination prevented further pharmacological exploration of KAR subunit composition in this study or investigation into the function of KARs in OPC biology. Future experiments with reporter mice, single-cell proteomics, or immunoprecipitation of KAR subunits could help define membrane surface expression of individual subunits, and association with auxiliary subunits, and provide a roadmap for specific knockout studies to investigate the role of KAR signaling in OPCs.

We also found that AMPAR current density increased with age, driven by a decrease in receptor desensitization and an increase in Ca^2+^ permeability. While modulating Ca^2+^ permeability has previously been shown to alter OPC proliferation and differentiation,^69^^,^^90^ we did not directly assess the role of altered desensitization in OPC fate. Rather, we provided evidence of regional and temporal diversity in AMPAR kinetics, which can now be leveraged to further investigate the impact of receptor desensitization using genetic tools.

## Resource availability

### Lead contact

Requests for further information and resources and reagents should be directed to, and will be fulfilled by, the lead contact, Ragnhildur T. Káradóttir (rk385@cam.ac.uk).

### Materials availability

This study did not generate new reagents.

### Data and code availability

• All data reported in this paper will be shared by the lead contact upon request.
• This paper does not report original code.
• Any additional information required to reanalyze the data reported in this paper is available from the lead contact upon request.

## Acknowledgments

We thank Prof. Jacqueline Trotter (Johannes Gutenberg-University, Mainz, Germany) for the NG2-EYFP mice, Dr. Sarah Crisp for providing hippocampal cultures, and Dr. Stavros Vagionitis for help with image processing. We thank members of the Káradóttir lab, Dr. Helena Pivoňková, and Dr. Ingo Greger for critical comments on the manuscript. We acknowledge the support of the Cambridge Stem Cell Institute core facility staff and University Biomedical Services. This project has received funding from the European Research Council (10.13039/100010663ERC) under the European Union’s Horizon 2020 research and innovation program (grant agreement No 771411; R.T.K, Y.K., K.A.E.); the 10.13039/100010269Wellcome Trust, a studentship (102160/Z/13/Z; Y.K.); MRC a program grant (MR/Y014537/1; R.T.K., K.A.E.); The Fonds de recherche du Québec-Santé, a scholarship (Y.K.); The Cambridge Commonwealth European & International Trust, a scholarship (Y.K.); MS Society Centre Excellence grant, Cambridge Myelin Repair Center grant (132; R.T.K., Y.T.N.); The Icelandic Research Fund (Rannis; No 206870-053, 2410652-051; R.T.K.); and the 10.13039/501100001255Lister Institute, a Research Prize (R.T.K.). This research was funded in whole, or in part, by the 10.13039/100010269Wellcome Trust (203151/Z/16/Z, 203151/A/16/Z) and the UKRI Medical Research Council (MC_PC_17230). For the purpose of open access, the author has applied a CC BY public copyright license to any Author Accepted Manuscript version arising from this submission.

## Author contributions

Conceptualization, R.T.K. and Y.K.; investigation, Y.K., K.A.E., and Y.T.N.; data analysis, Y.K., K.A.E., S.D., and R.T.K.; writing, Y.K. and R.T.K., and all authors commented on the manuscript; funding acquisition, resources, and supervision, R.T.K.

## Declaration of interests

The authors declare no competing interests.

## STAR★Methods

### Key resources table

**Table:** 

REAGENT or RESOURCE	SOURCE	IDENTIFIER
**Antibodies**		

Chicken anti-GFP	Abcam	Cat#ab13970; RRID:AB_300798
Rabbit anti-Olig2	Millipore	Cat#AB9610; RRID:AB_570666
Goat Anti-Chicken IgY H&L (Alexa Fluor 568)	Abcam	Cat#ab175477; RRID:AB_3076392
Invitrogen Goat anti-Rabbit IgG (H + L) Highly Cross-Adsorbed Secondary Antibody, Alexa Fluor 647	ThermoFisher Scientific	Cat#A-21245; RRID:AB_2535813
Rabbit anti-GluK1	Synaptic Systems	Cat#180 313 RRID:AB_2884928
Rabbit anti-GluR6	Synaptic Systems	Cat#180 003 RRID:AB_2114166
Rabbit anti-GRIK3	Alomone Labs	Cat#AGC-040 RRID:AB_2340955
Rabbit anti-GRIK4	Alomone Labs	Cat#AGC-041 RRID:AB_2340956
Rabbit anti-KA2	Synaptic Systems	Cat#180 103 RRID:AB_2111993
Mouse anti-β-actin	Sigma-Aldrich	Cat#A5441 RRID:AB_476744
IRDye 800CW Donkey anti-Rabbit IgG	LI-COR Biosciences	Cat#926-32213 RRID:AB_621848
IRDye 680RD Donkey anti-Mouse IgG	LI-COR Biosciences	Cat#926-68072 RRID:AB_10953628

**Chemicals, peptides, and recombinant proteins**		

NaCl	Sigma-Aldrich	Cat#S7653
KCl	Sigma-Aldrich	Cat#P3911
NaHCO_3_	Sigma-Aldrich	Cat#S5761
NaH_2_PO_4_	Fisher Scientific	Cat#S/3760/53
CaCl_2_	VWR	Cat#21114
MgCl_2_	Fisher Scientific	Cat#15656060
D-glucose	Sigma-Aldrich	Cat#G7528
Kynurenic acid	Sigma-Aldrich	Cat#K3375
HEPES	Sigma-Aldrich	Cat#H3375
NaOH	Sigma-Aldrich	Cat#06203
D-gluconic acid	Sigma-Aldrich	Cat#G1951
CsOH	Sigma-Aldrich	Cat#516988
MgATP	Sigma-Aldrich	Cat#A9187
Na_2_GTP	Sigma-Aldrich	Cat#G8877
K-Lucifer Yellow	Sigma-Aldrich	Cat#L0144
Alexa Fluor 594 Hydrazide	ThermoFisher Scientific	Cat#A10438
BaCl_2_	Sigma-Aldrich	Cat#B0750
SYM2081	Tocris	Cat#2081
Concanavalin A	Sigma-Aldrich	Cat#L7647
UBP301	Tocris	Cat#1766
Goat serum	Sigma-Aldrich	Cat#G9023
Triton X-100	Sigma-Aldrich	Cat#T9284
DAPI	Sigma-Aldrich	Cat#D9542
Fluoromount	Cambridge Bioscience	Cat#0100-01
Neurobasal medium	Gibco	Cat#21103049
B27 supplement	Gibco	Cat#17504001
Penicillin/Streptomycin	Sigma	Cat#P0781
Glutamax	Life Technologies	Cat#35050038
RIPA buffer	EMD Millipore	Cat#20-188
DNAse	Sigma-Aldrich	Cat#D5025-150KU
Intercept (TBS) Blocking Buffer	LI-COR Biosciences	Cat#927-60001
Tween 20	Sigma-Aldrich	Cat#P9416
Quick Start™ Bradford 1x Dye Reagent	Bio-Rad	Cat#500-0205

**Critical commercial assays**		

Myelin Removal Beads II, human, mouse, rat	Miltenyi Biotec	Cat# 130-096-733
CD140a (PDGFRα) MicroBead Kit, mouse	Miltenyi Biotec	Cat# 130-101-502

**Deposited data**		

OPC bulk RNA sequencing	Spitzer et al., 2019^20^	GEO: GSE121083
OPC single-cell RNA sequencing	Heo et al., 2025^47^	GEO: GSE249268

**Experimental models: Organisms/strains**		

Mouse: NG2-EYFP	Jacqueline Trotter; Karram et al., 2008^34^	N/A
Rat: Crl:CD(SD)	Charles River Laboratories	RRID:RGD_734476

**Software and algorithms**		

pClamp	Molecular Devices	https://www.moleculardevices.com/products/axon-patch-clamp-system/acquisition-and-analysis-software/pclamp-software-suite
LAS X	Leica	https://www.leica-microsystems.com/products/microscope-software/details/product/leica-las-x-ls/
FIJI	NIH	https://fiji.sc/or https://imagej.nih.gov/ij/
MATLAB	The MathWorks	https://uk.mathworks.com/
Membrane capacitance and resistance script	Spitzer et al., 2019^20^	N/A
Voltage ramp analysis script	This paper	N/A
GraphPad Prism	GraphPad Software	https://www.graphpad.com/scientific-software/prism/

### Experimental model and study participant details

Experiments were performed in accordance with EU guidelines for the care and use of laboratory animals, and with the guidelines of the UK Animals (Scientific Procedures) Act 1986 and subsequent amendments. Use of animals in this project was approved by the Animal Welfare and Ethical Review Body for the University of Cambridge and carried out under the terms of UK Home Office Licenses P9B1FBC4B and PP4353554.

#### Mice

All mice were maintained under a 12h light:12h dark cycle with food and water supplied *ad libitum*. To identify OPCs in acute brain slices, we used both male and female heterozygous knock-in NG2-EYFP transgenic mice,^34^ kindly donated by Jacqueline Trotter, in which NG2^+^ cells express EYFP. To identify a kainate concentration specific to KARs, OPCs were recorded in P14-22 mice. To test KAR-specific drugs, OPCs were recorded in P13-15, P20-25, P40-58, P99, and P182 mice. To examine changes in AMPAR and KAR current densities, OPCs were recorded at the following ages: E13; E17; P7: P7-8; 1 month: P22-36; 3 months: P100-130; 7 months: P210-P230; 12 months: P355-357. Current densities did not differ between male and female mice.

#### Hippocampal neuron cultures

Hippocampi were dissected from E18 rat embryos and cells were dissociated with Neuron Isolation Enzyme (Thermo Scientific; Cat no. 88285). 50K cells were plated on poly-D-lysine coated coverslips in 24-well plates, in neurobasal medium (Gibco; Cat. no. 21103049) supplemented with B27 (Gibco; Cat. no. 17504001), glutamax (Life Technologies; Cat. no. 35050038), and penicillin-streptomycin (Sigma; Cat. no. P0781). A half-media change was performed twice per week. Cells were used for electrophysiological recordings on 17–20 days *in vitro* (DIV).

### Method details

#### Acute brain slices

225 μm-thick coronal slices (to preserve axonal integrity and optimize cell visualization) were cut from NG2-EYFP mice between P7 and P230 in ice-cold (∼1 °C) oxygenated (5% CO_2_/95% O_2_) bicarbonate-buffered artificial cerebrospinal fluid (aCSF) containing, in mM: 124 NaCl, 26 NaHCO_3_, 1 NaH_2_PO_4_, 2.5 KCl, 2 MgCl_2_, 2.5 CaCl_2_, 10 glucose, pH 7.4, 330 mOsm. 1 mM kynurenic acid was added to block glutamate receptors that might be activated during dissection. Embryonic brains (E13 and E17) were embedded in 2% agarose prepared in HEPES-buffered solution containing, in mM: 144 NaCl, 10 HEPES, 1 NaH_2_PO_4_, 2.5 KCl, 2 MgCl_2_, 2.5 CaCl_2_, and 10 glucose and sliced in 225–325 μm-thick coronal sections as above.^96^

#### Electrophysiology

All experiments were performed in whole-cell voltage-clamp mode, with junction potential (−14 mV) compensated holding potential −74 mV. Pipette resistance was between 4.3 and 6.5 MΩ and mean uncompensated series resistance was 25 MΩ. Recordings were performed in HEPES-buffered aCSF containing, in mM: 144 NaCl, 10 HEPES, 1 NaH_2_PO_4_, 2.5 KCl, 1–2 MgCl_2_, 2.5 CaCl_2_ and 10 glucose, with pH adjusted to 7.2–7.4 with 1 M NaOH, and osmolarity 315 mOsm. For experiments with cyclothiazide (CTZ) or PEPA, 0.1% DMSO was added to the external solution, as a solvent control. For experiments with UBP301, 1% DMSO was added to the external solutions, as a solvent control. The internal solution contained, in mM: 130 Cs-gluconate, 4 NaCl, 0.5 CaCl_2_, 10 HEPES, 10 BAPTA, 4 Mg_x_ATP, 0.5 Na_x_GTP, and 2 K-Lucifer Yellow or 0.001 Alexa Fluor 594, pH adjusted to 7.2–7.4 with 2 M CsOH, with osmolarity between 290 and 300 mOsm. All recordings took place at room temperature, and the recording solution was continuously oxygenated with 100% O_2_. Inclusion criteria was based on series resistance, leak current being smaller than 400 pA and a stable baseline. In addition, cells that exhibited both ohmic passive currents and tail currents following a voltage step protocol were excluded as this is indicative of immature oligodendrocytes^93^; these represent <1.5% of NG2-EYFP cells. An Axopatch 200 (Molecular Devices) was used for voltage-clamp data acquisition. Voltage ramp data were sampled at 50 kHz and filtered at 10 kHz and drug application data were sampled at 1 kHz using pClamp 10.7 or pClamp 11 (Molecular Devices). Cells were recorded in the anterior corpus callosum and cortical layers 2–6. During recordings, cells were filled with Lucifer Yellow or Alexa Fluor 594. Location and cell identity were confirmed by post-hoc immunohistochemistry against GFP. In 26/26 cases, imaged cells were positive for EYFP, and 11/11 imaged cells were positive for EYFP and OLIG2. Using this method, we have found that >98% of recorded NG2-EYFP cells express the native NG2 protein, and that 100% express OLIG2.

#### Drugs

3-30 μM kainate was used to activate both AMPARs and KARs, or KARs only, as specified in the results. 10 μM GYKI53655 was used to specifically block AMPARs.^37^ 10 μM SYM2081 was used to activate KARs,^33^ and 0.3 mg/mL Concanavalin A was used to alter KAR currents.^42^ As previously described, Concanavalin A was applied for 8 min prior to kainate application.^42^^,^^48^^,^^58^ 50 μM UBP301 was used to specifically block KARs.^59^ 10 μM AMPA was used to activate AMPARs. 25 μM–100 μM CTZ and 25 μM–100 μM PEPA, as specified in the results, were used to measure the relative abundance of flip and flop AMPAR subunit variants.^78^^,^^80^ When recording from OPCs, 200 μM BaCl_2_ was added to the recording solution to block inward K^+^ conductance.^20^^,^^23^^,^^96^^,^^97^

#### Electrophysiological analysis

Voltage ramps ranging from −134 mV to 26 mV were applied before, at the peak of the steady state response, and following recovery from AMPA application. To isolate AMPA-evoked currents, the baseline-corrected mean of the pre-application and post-recovery ramps was subtracted from the peak response ramp and the rectification index (RI) was calculated as $=\frac{I_{+20mV}-I_{rev}}{I_{rev}-I_{-70mv}}$ . The reversal potential (E_AMPA_) was measured by fitting the voltage ramp to a linear curve between −34 mV and 26 mV and calculating the potential at *I*_*rev*_ = 0 pA. −14 mV were added to compensate for junction potential. Both RI and E_AMPA_ were calculated using a custom written MATLAB script (MathWorks). Series resistance and membrane capacitance were calculated as previously described^96^^,^^98^ using a custom written MATLAB script.^20^

#### Post-hoc immunohistochemistry and imaging

Post-hoc immunohistochemistry was performed as previously described.^96^^,^^99^ Briefly, slices were incubated in 4% PFA for 1 h at room temperature, before being washed in PBS. For antibody labeling, slices were incubated in 10% goat serum and 0.5% Triton X-100 in PBS for 4–5 h at room temperature, on a rotating shaker. Slices were incubated with primary antibodies in PBS overnight at room temperature. Primary antibodies were as follows: chicken anti-GFP, 1:1000 (Abcam, ab13970) and rabbit anti-OLIG2, 1:300 (EMD Millipore, AB9610). Following three 30 min washes in PBS, the slices were incubated in secondary antibodies in PBS at a 1:1000 concentration overnight at 4°C or for 5 h at room temperature, on a rotating shaker. Secondary antibodies were as follows: goat anti-chicken IgY Alexa Fluor 448 (Abcam, ab150169), goat anti-chicken IgY Alexa Fluor 568 (Invitrogen, A-11041) and goat anti-rabbit IgG Alexa Fluor 647 (Invitrogen, A-21245). After two washes in PBS, slices were incubated with 1 ng/mL DAPI for 20 min, and following a final wash, mounted on glass slides.

Samples were imaged on a Leica TCS SP5 microscope or a Leica TCS SP8 microscope. Laser intensity, voltage and offset were adjusted to maximise the signal to noise ratio. Parameters were kept constant for negative control slices. Images were acquired at 600Hz and frame averaged 2–4 times, as needed. Images of patched cells were obtained on a 63× oil objective. z stack thickness depended on cell morphology, and was between 5 and 20 μm. Optical slice thickness was 0.5 μm. Images were visualised and processed in LAS X and Arivis Vision 4D.

#### Western blots

Mouse OPCs were isolated from stock transgenic mice at P6, P30, and P52-54 by Magnetic Activated Cell Sorting (Miltenyi Biotec), as previously described,^20^^,^^96^ and following the manufacturer’s instructions. Briefly, myelin debris were removed with myelin removal beads (Cat. no. 130-096-733, Miltenyi Biotec) and OPCs were then isolated with CD140-conjugated microbeads (Cat. no. 130-101-502, Miltenyi Biotec). The isolated OPCs, cultured dorsal root ganglia neurons,^100^ E14.5 whole brains, and P52 or P500 cerebellums were mechanically homogenized in RIPA buffer (Cat. no. 20–188, EMD Millipore) with 5% DNAse (D5025-150KU, Sigma-Aldrich). Sample protein concentration was determined by Braford assay (Cat. no. 500-0205, Bio-Rad). 30 μg from samples were loaded on a NuPAGE 4–12% Bis-Tris gel (Cat. no. NP0322PK2, Thermo Fisher Scientific) for protein separation by electrophoresis. Proteins were transferred onto a nitrocellulose membrane using a wet transfer system for 90 min. Following protein transfer, the membranes were blocked in Intercept (TBS) Blocking Buffer (Cat. no. 927–60001, LI-COR Biosciences) with 0.1% Tween 20 for 1 h at room temperature. Membranes were incubated in primary antibodies in blocking buffer overnight at 4°C. The following antibodies were used: rabbit anti-GluK1 (1:1000; Cat. no. 180 313, Synaptic Systems), rabbit anti-GluR6 (1:1000; Cat. no. 180 003, Synaptic Systems), rabbit anti-GRIK3 (1:300; Cat. no. AGC-040, Alomone Labs), rabbit anti-GRIK4 (1:400; Cat. no. AGC-041, Alomone Labs), rabbit anti-KA2 (1:1000; Cat. no. 180 103, Synaptic Systems), and mouse anti β-actin (1:10000; Cat. no. A5441, Sigma-Aldrich). Following this, membranes were washed three times (20 min each) in 0.1% Tween 20 in PBS and incubated with secondary antibodies for 1 h at room temperature. The following antibodies were used: donkey anti-rabbit 800 (1:10000; Cat. no. 926–32213, LI-COR Biosciences) and donkey anti-mouse 680 (1:10000; Cat. no. 926–68072, LI-COR Biosciences). Following this, membranes were washed three times (20 min each) and imaged on an LI-COR Odyssey Fc.

### Quantification and statistical analysis

Data are shown as mean ± SEM. Statistics were computed in GraphPad Prism or manually in Excel. When comparing two conditions, unpaired two-tailed t-tests were used; variance was tested by F-test, and Welch’s correction applied if unequal. When comparing data normalized to control, one sample t-tests were used. Proportions were tested with a χ^2^ test, with Yates’ correction for small numbers. When comparing three or more conditions, one-way ANOVA were used; variance was tested with a Brown-Forsythe test, and Welch’s correction was applied if variance was unequal. Post-hoc comparisons were performed with Holm-Bonferroni tests. Statistical significance of changes in AMPAR and KAR subunit transcripts with age was previously assessed in Spitzer et al., 2019^20^ using the R Bioconductor DESeq2 package. Statistical significance across AMPAR and KAR subunit transcripts was assessed by Dunn’s test of multiple comparisons with Bonferroni correction following a significant (*p* < 0.05) Kruskal-Wallis test. The single-cell sequencing OPC dataset was obtained from GEO (accession number: GSE249268) and OPC states were reanalysed with Scanpy using default parameters. Differential expression between OPC states was evaluated in Scanpy using the Wilcoxon rank-sum test. P-values were adjusted for multiple testing using the Benjamini-Hochberg False Discovery Rate. ∗ indicates *p* ≤ 0.05. Statistical details for individual experiments can be found in the figures and their legends.
