# Supplementary material for: Oligodendrocyte precursor cell AMPA receptors differ with age and brain region while kainate receptors remain stable
Source: iScience. 2025 Sep 13;28(10):113560. doi: 10.1016/j.isci.2025.113560 (PMC12509198; doi:10.1016/j.isci.2025.113560)
Supplement: Document S1. Figures S1–S6 [file mmc1.pdf]

iScience, Volume *28*

Supplemental information

Oligodendrocyte precursor cell AMPA receptors differ with age and brain region while kainate receptors remain stable

Yasmine Kamen, Kimberley Anne Evans, Yan Ting Ng (吳胤霆), Sabine Dietmann, and Ragnhildur Thóra Káradóttir

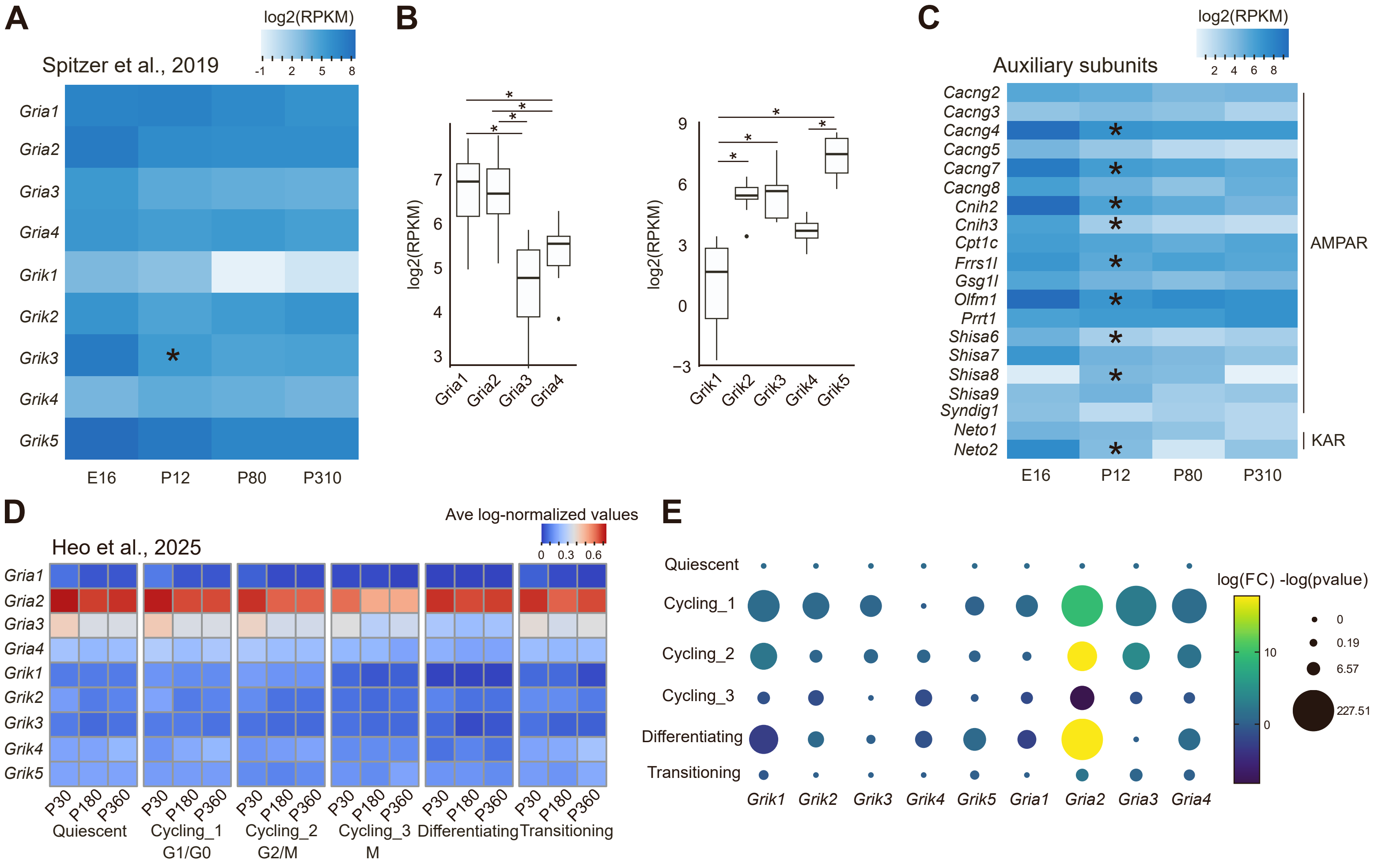

Figure S1, related to Figure 1. OPCs express *Gria* and *Grik* transcripts

A-C Bulk RNA sequencing of whole brain OPCs from Spitzer et al., 2019[20]. **A** Mean AMPAR and KAR subunit transcript levels across age. AMPAR and KAR subunit transcript levels do not differ with postnatal age. The asterisk indicates the only statistically significant (p≤0.05) difference when testing individual subunits across age, with *Grik3* reduced between E16 and P12 (n=3-4 per timepoint). Statistical significance was previously assessed in Spitzer et al., 2019[20] using the R Bioconductor DESeq2 package. **B** Expression level for AMPAR (top) and KAR (bottom) subunits pooled across postnatal ages. The horizontal line indicates the median, the boxes extend from the 25th to 75th percentile, and the vertical lines indicate the range, with outliers indicated by individual points. The asterisks indicate statistically significant (p≤0.05) differences between AMPAR or KAR subunits (n=3 per subunit; Kruskall-Wallis test followed by Dunn's test of multiple comparisons with Bonferroni correction). **C** Mean AMPAR and KAR auxiliary subunit transcript levels across age. AMPAR and KAR auxiliary subunit transcript expression does not differ postnatally. The asterisks in the heat chart indicate statistically significant changes (p≤0.05), which all occur between E16 and P12 (n=3-4 per timepoint). Statistical significance was previously assessed in Spitzer et al., 2019[20] using the R Bioconductor DESeq2 package. **D-E** Single-cell RNA sequencing of cortical OPCs from Heo et al., 2025[47]. **D** Average log-normalized AMPAR and KAR subunit transcripts with age and OPC state. AMPAR and KAR subunit transcripts are similar across age and cell states. **E** Bubble plot showing the log(Fold change) (colour scale) of *Grik* and *Gria* transcripts in different OPC states (all ages pooled) when compared to quiescent OPCs. The size of individual bubbles indicates the -log(pvalue), as calculated by the Wilcoxon rank-sum test with p values adjusted for multiple comparisons with the Benjamini–Hochberg False Discovery Rate.

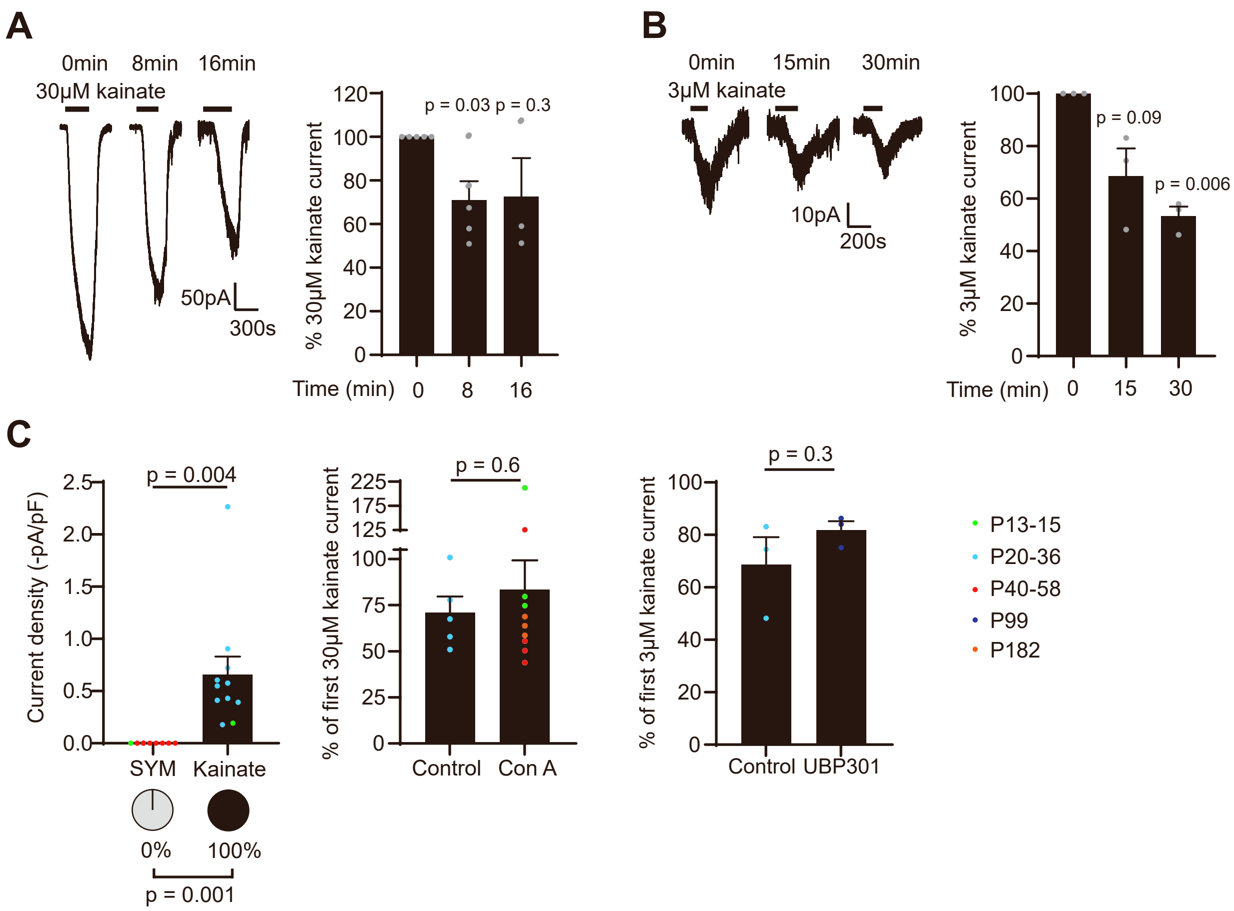

Figure S2, related to Figure 1. Kainate-evoked currents run down with time in OPCs
A 30 μM kainate-evoked currents run down with time during repeated applications. n=3-5 cells from 2 mice. **B** 3 μM kainate-evoked currents run down with time during repeated applications. n=3 cells from 1 mouse. **C** Data from Figure 1D (left), 1F (middle), 1H (right) are shown with individual data points coloured according to the age of the mouse the cell was recorded from. Data are shown as mean±SEM, with dots indicating individual recorded cells. Statistics were calculated by one sample t-test (A, B) and unpaired two-tailed t-test (C).

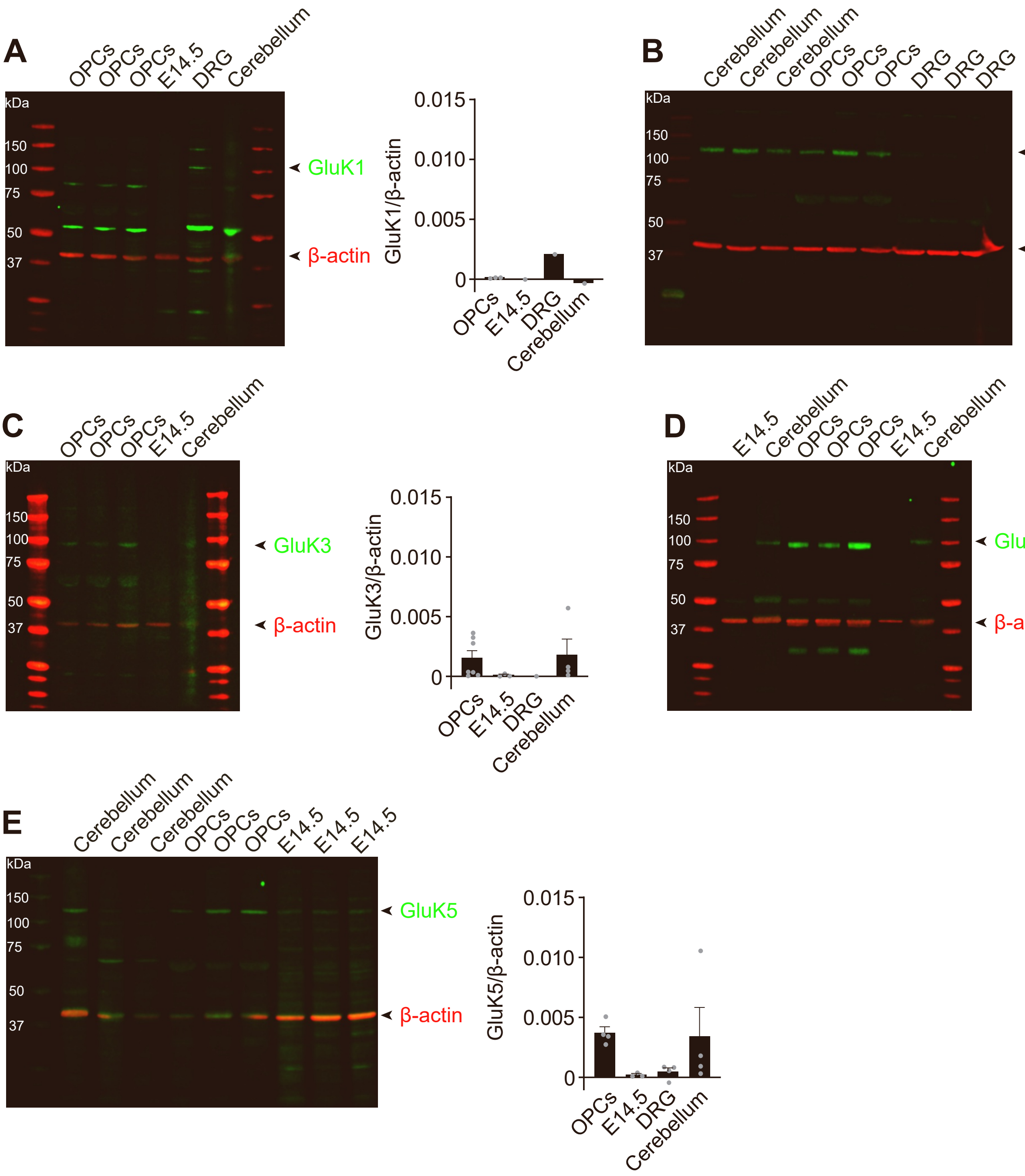

Figure S3, related to Figure 1. OPCs express GluK2-5 protein

A-E Western blots and fluorescence intensity quantification for GluK1 (A), GluK2 (B), GluK3 (C), GluK4 (D), and GluK5 (E) in P6, P30, or P52-54 OPCs (n=3-7), E14.5 whole brain homogenates (n=1-3), cultured rat dorsal root ganglia neurons (DRG; n=1-4), and P52 or P500 cerebellum homogenates (n=1-4). E14.5 whole brain homogenates were used as negative controls for all subunits[S1]. Cultured rat dorsal root ganglia neurons were used as a positive control for GluK1 and a negative control for GluK2, GluK3, and GluK4[41,42]. P52 or P500 cerebellar homogenates were used as a negative control for GluK1 and positive controls for all other subunits[S2],[S3]. Fluorescence intensity analysis suggests that OPCs do not have detectable GluK1, but have clear detectable bands at the expected molecular weights for GluK2, GluK3, GluK4, and GluK5. Grey dots indicate individual protein samples, averaged from 1-3 technical replicates. Data are shown as mean±SEM.

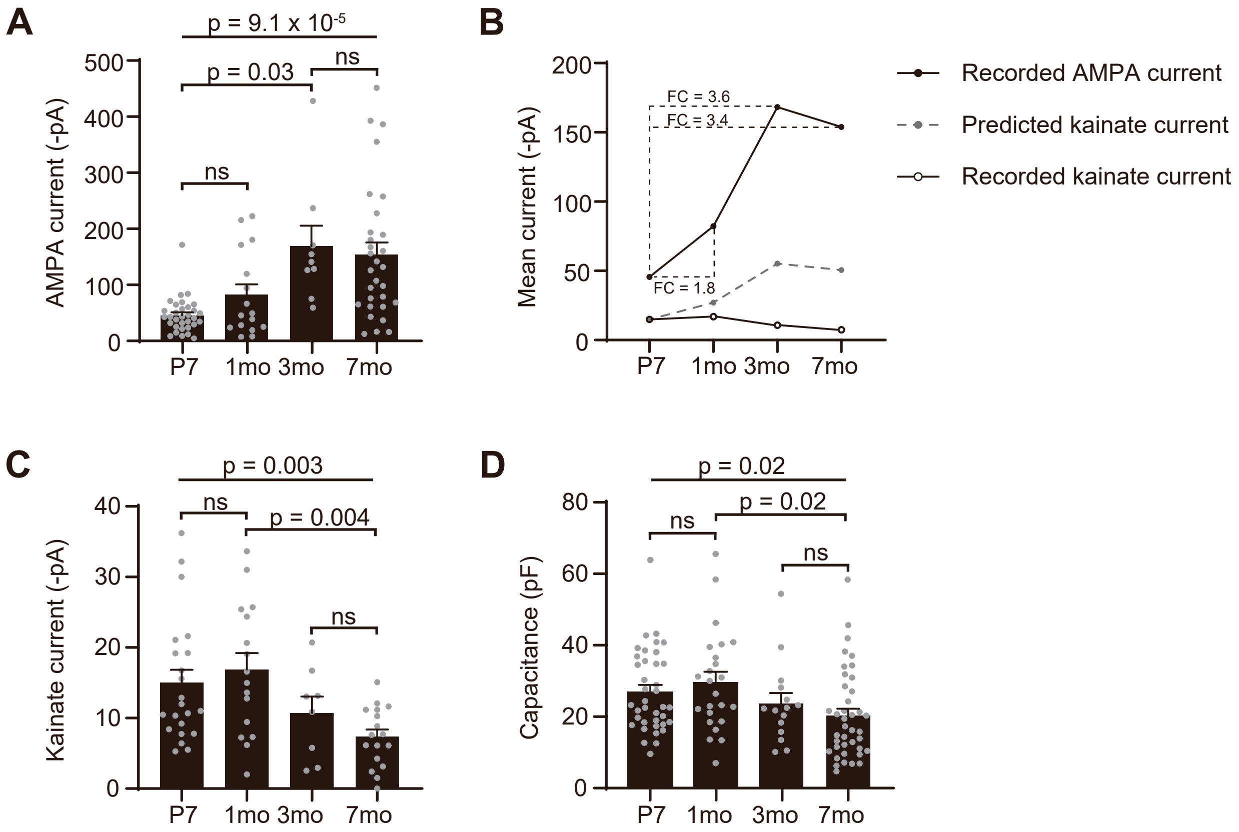

Figure S4, related to Figure 2. 3 µM kainate does not activate AMPARs

A 10 µM AMPA-evoked currents increased with age in OPCs in the cortex and corpus callosum. P7: n=32 cells from 7 mice; 1 mo: n=16 cells from 4 mice ; 3 mo: n=9 cells from 4 mice; 7 mo: n=30 cells from 9 mice. **B** Plotting mean recorded 10 µM AMPA-evoked currents with age (black circles) allows the calculation of the fold change (FC) between P7 and 1 month (mo; (1.8), P7 and 3 mo (3.6), and P7 and 7 mo (3.4). If 3 µM kainate-evoked currents activated AMPAR, a similar fold change in mean current would be expected; predicted mean currents were calculated by multiplying the recorded 3µM kainate-evoked current at P7 by the fold changes calculated in mean AMPA-evoked currents (grey circles). Mean recorded 3 µM kainate-evoked currents (open circles) differed from the predicted currents. **C** 3 µM kainate-evoked currents decreased with age in OPCs in the cortex and corpus callosum. P7: n=22 cells from 5 mice; 1 mo: n=16 cells from 4 mice; 3 mo: n=8 cells from 4 mice; 7 mo: n=17 cells from 8 mice. **D** Cell membrane capacitance differs with age in OPCs in the cortex and corpus callosum. P7: n=38 cells from 7 mice; 1 mo: n=24 cells from 4 mice; 3 mo: n=15 cells from 4 mice; 7 mo: n=40 cells from 9 mice. Data are shown as mean±SEM, with grey dots indicating individual recorded cells. Statistics were calculated by ANOVA (p-values above) and Holm-Bonferroni post-hoc tests (p-values below).

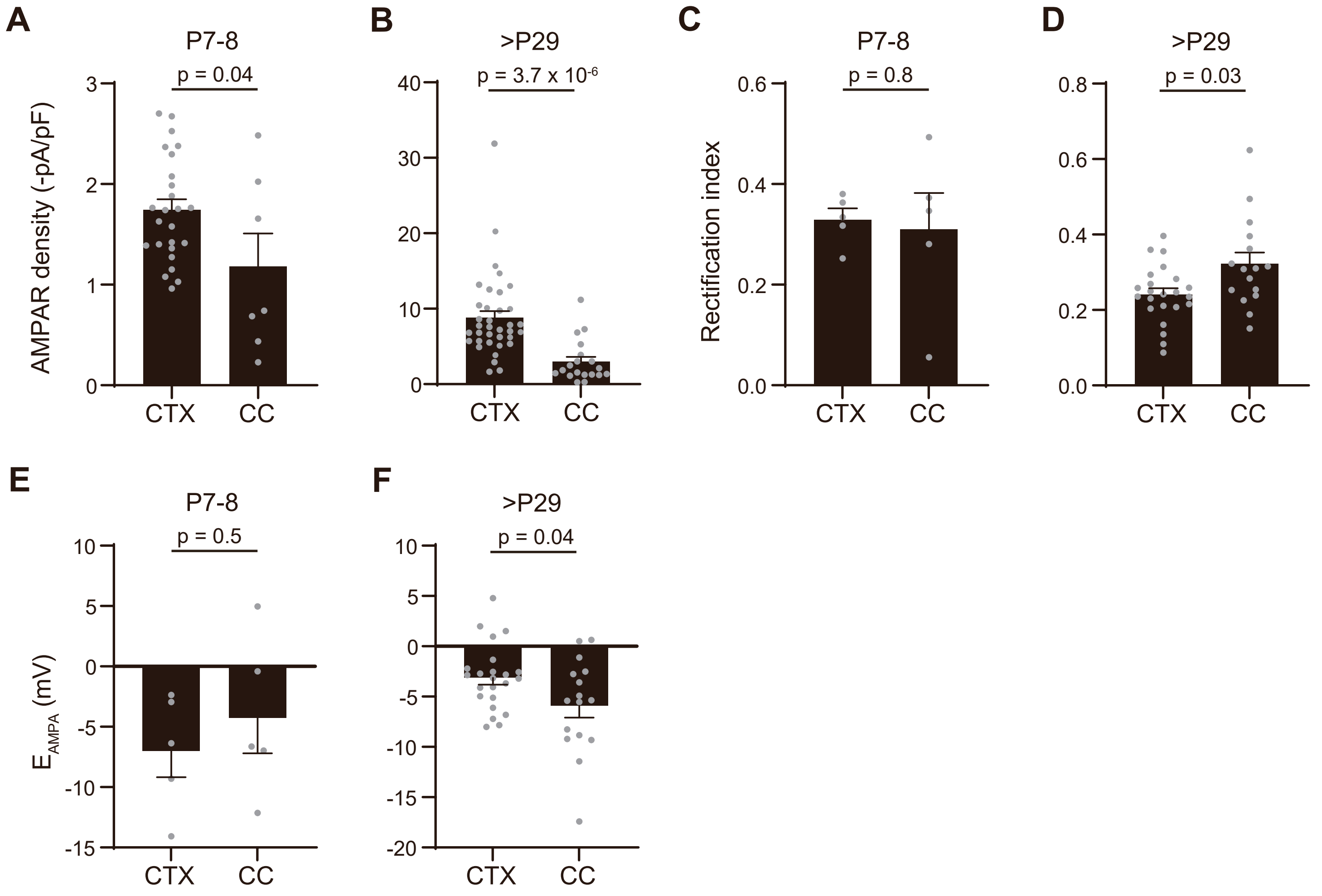

Figure S5, related to Figures 3 and 4. AMPAR current density differs between grey and white matter

A AMPAR density was higher in the cortex (CTX) compared to the corpus callosum (CC) at P7-8. CTX: n=25 cells from 7 mice; CC: n=7 cells from 3 mice. **B** AMPAR density remained higher in the CTX compared to the CC after P29. CTX: n=36 cells from 16 mice; CC : n=19 cells from 10 mice. **C** The rectification index does not differ between cortical and callosal OPCs at P7-8. CTX: n=5 cells from 3 mice; CC: n=5 cells from 3 mice. **D** The rectification index was lower in the CTX compared to the CC after P29. CTX: n=23 cells from 12 mice; CC : n=16 cells from 10 mice. **E** The reversal potential (E_{AMPA}) did not differ between the CTX and CC at P7-8. CTX: n=5 cells from 3 mice; CC: n=5 cells from 3 mice. **F** After P29, E_{AMPA} became less negative in the CTX than in the CC. CTX: n=23 cells from 12 mice; CC : n=16 cells from 10 mice. Data are shown as mean±SEM, with grey dots indicating individual recorded cells. Statistics were calculated with unpaired two-tailed t-tests.

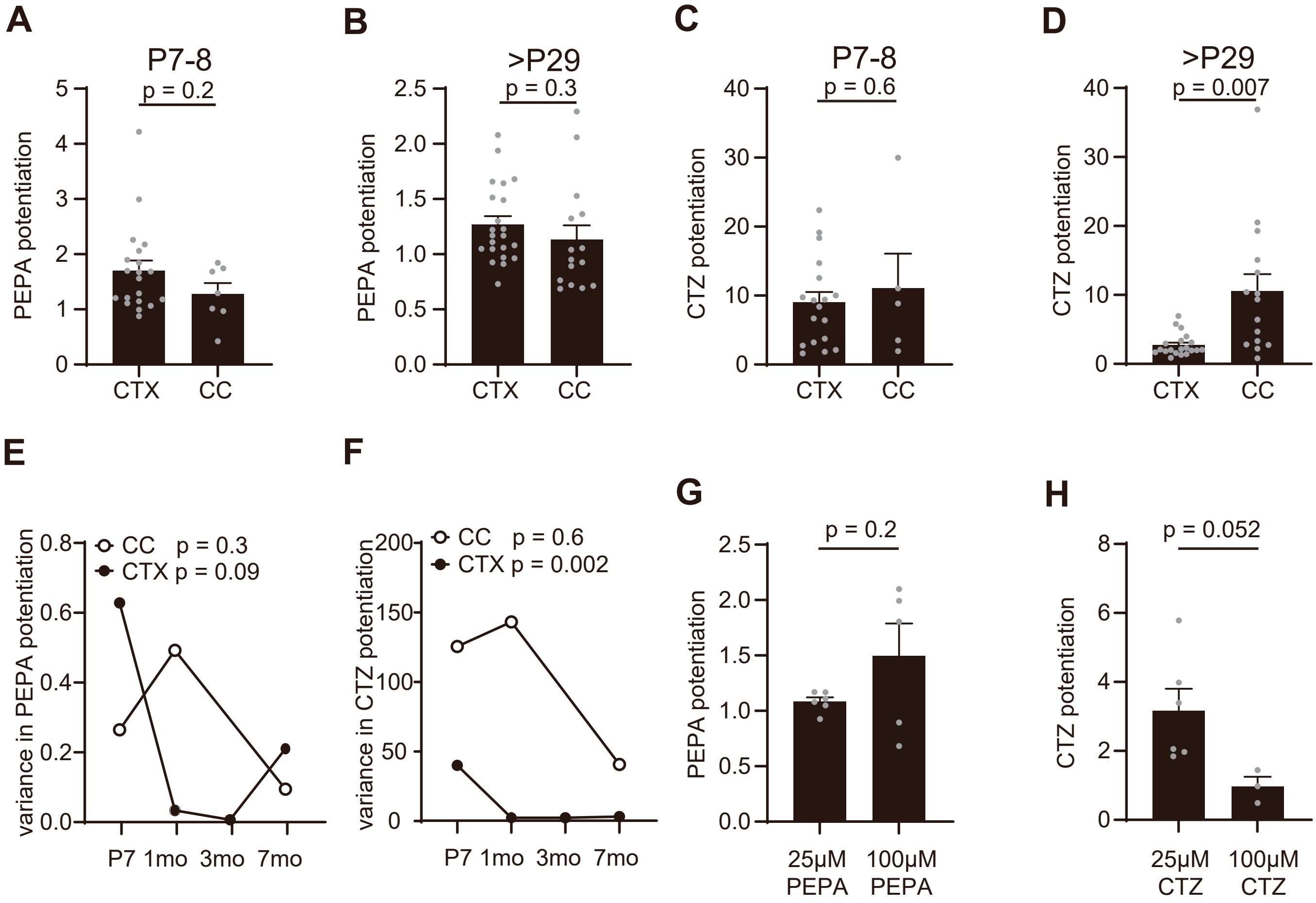

Figure S6, related to Figure 5. AMPAR subunits may differ between regions

A PEPA potentiation of AMPA-evoked currents did not differ between the cortex (CTX) and corpus callosum (CC) at P7-8. CTX: n=20 cells from 5 mice; CC: n=7 cells from 3 mice. **B** PEPA potentiation of AMPA-evoked currents did not differ between the CTX and CC after P29. CTX: n=22 cells from 14 mice; CC: n=15 cells from 9 mice. **C** CTZ potentiation of AMPA-evoked currents did not differ between the CTX and CC at P7-8. CTX: n=18 cells from 5 mice; CC: n=5 cells from 3 mice. **D** CTZ potentiation of AMPA-evoked currents was lower in the CTX than in the CC after P29. CTX: n=20 cells from 11 mice; CC: n=15 cells from 9 mice. **E** Variance in PEPA potentiation of AMPA-evoked currents did not differ with age in the CTX or the CC. CTX: P7: n=20 cells from 5 mice; 1 mo: n=5 cells from 3 mice; 3 mo: n=6 cells from 2 mice; 7 mo: n=11 cells from 7 mice. CC: P7: n=7 cells from 3 mice; 1 mo: n=6 cells from 3 mice; 7 mo: n=9 cells from 6 mice. **F** Variance in CTZ potentiation of AMPA-evoked currents decreased with age in the CTX, but did not differ with age in the CC. P7: n=18 cells from 5 mice; 1 mo: n=5 cells from 3 mice; 3 mo: n=6 cells from 2 mice; 7 mo: n=9 cells from 6 mice. CC: P7: n=5 cells from 3 mice; 1 mo: n=6 cells from 3 mice; 7 mo: n=9 cells from 6 mice. **G** Potentiation of AMPA-evoked currents by 100 µM PEPA did not differ from potentiation of AMPA-evoked currents by 25 µM PEPA in 3 months (mo) cortical OPCs. 25 µM: n=6 cells from 2 mice; 100 µM: n=5 cells from 2 mice. **H** Potentiation of AMPA-evoked currents by 100 µM CTZ did not differ from potentiation of AMPA-evoked currents by 25 µM CTZ in 3 mo cortical OPCs. 25 µM: n=6 cells from 2 mice; 100µM: n=3 cells from 2 mice.Data are shown as mean±SEM, with grey dots indicating individual recorded cells. Statistics were calculated by unpaired two-tailed t-tests (A-D), Brown-Forsythe tests (E, F) or ANOVA (p-values above) with Holm-Bonferroni post-hoc tests (p-values below) (G, H).

Supplemental References

[S1] Bahn, S., Volk, B., and Wisden, W. (1994). Kainate receptor gene expression in the developing rat brain. J Neurosci *14*, 5525–5547. 10.1523/JNEUROSCI.14-09-05525.1994.

[S2] Watanabe-Iida, I., Konno, K., Akashi, K., Abe, M., Natsume, R., Watanabe, M., and Sakimura, K. (2016). Determination of kainate receptor subunit ratios in mouse brain using novel chimeric protein standards. J Neurochem *136*, 295–305. 10.1111/jnc.13384.

[S3] Ripellino, J.A., Neve, R.L., and Howe, J.R. (1997). Expression and heteromeric interactions of non-N-methyl-D-aspartate glutamate receptor subunits in the developing and adult cerebellum. Neuroscience *82*, 485–497. 10.1016/S0306-4522(97)00296-0.
